# Pulmonary vasoreactive reserve in patients with idiopathic pulmonary arterial hypertension: characteristics and clinical significance

**DOI:** 10.3389/fcvm.2026.1893751

**Published:** 2026-10-14

**Authors:** Natalia Goncharova, Kirill Lapshin, Aelita Berezina, Artem Isakov, Aleksandra Vatian, Anton Ryzhkov, Irina Zlobina, Zaneta Matakaeva, Elizaveta Korobchenko, Olga Moiseeva

**Affiliations:** 1 Almazov National Medical Research Center Ministry of Health of Russia, Saint-Petersburg, Russia; 2 Information Technologies, Mechanics and Optics University, Saint Petersburg, Russia

**Keywords:** acute vasoreactive testing, idiopathic pulmonary arterial hypertension, prognosis, pulmonary artery compliance, risk stratification, pulmonary vasoreactive reserve

## Abstract

**Introduction:**

We hypothesized that differences in pulmonary hemodynamics during acute vasoreactive testing (AVT), beyond the conventional Sitbon criteria, may be described as pulmonary vasoreactive reserve (VRR) that could be used to assess disease severity and prognosis in idiopathic pulmonary arterial hypertension (IPAH) patients. The study aimed to evaluate the clinical significance of VRR in patients with IPAH.

**Methods:**

Ninety adult IPAH patients (40.4 ± 14.6 years, 14.3% males) from a prospective single-center PAH registry were divided into three groups based on mean pulmonary artery pressure (mPAP) decrease during AVT: Group I (*n* = 25): ΔmPAP > 30%; Group II (*n* = 13): ΔmPAP 15%–30%; and Group III (*n* = 52): ΔmPAP < 15%. A comparison was performed between the standard model of survival prediction using the 2022 European Society of Cardiology/European Respiratory Society (ESC/ERS) three-strata risk stratification scale and the experimental model with ΔmPAP grading.

**Results:**

The absolute value of ΔmPAP was 21 mmHg (14; 28) in Group I, 11 mmHg (10; 12) in Group II, and only 2.5 mm Hg (1; 4.5) in Group III. The Δ%mPAP significantly differed in low-, intermediate-, and high-risk 2022 ESC/ERS three-strata categories (32.4 (12.3; 42.2) vs. 12.9 (3.01; 28.2) vs. 4.8% (1.8; 8.9), *p* = 0.0009). Significant association was revealed between the Δ%mPAP and pulmonary artery compliance (*r* = 0.30; *p* = 0.0045), pulmonary artery distensibility (*r* = 0.37, *p* = 0.0058), and cardiopulmonary exercise test parameters. During a follow-up of 2.32 years (1.04; 4.06), 17 (18.8%) patients died. The survival rate was 93%, 83%, 78%, 62%, and 55% at 1, 3, 5, 7, and 10 years, respectively. The degree of Δ%mPAP [heart rate (HR) 5.99 (1.37–26.2), *p* = 0.017], N-terminal pro-brain-type natriuretic peptide > 1,100 pg/mL [HR 8.1 (1.67–39.2), *p* = 0.009], and the number of PAH-specific drugs [HR 0.07 (0.018–0.3), *p* = 0.0003] were considered independent predictors of survival. The inclusion of Δ%mPAP slightly improved prognostic value for the 2022 ESC/ERS three-strata risk stratification scale in comparison with the standard 2022 ESC/ERS three-strata approach (mean absolute percentage error, MAPE = 9.66%; C-index = 0.7552742616033755 vs. MAPE = 9.82%; C-index = 0.7510548523206751).

**Conclusion:**

The assessment of VRR may warrant further investigation as an additional marker of the disease severity and prognosis in IPAH patients.

## Introduction

Right ventricular (RV) heart failure is the leading cause of mortality in patients with pulmonary arterial hypertension (PAH). Reverse right-heart remodeling due to the pulmonary artery pressure (PAP) decrease is an expected treatment effect of pulmonary vasodilators. The vasodilatory properties of pulmonary arteries determine the effectiveness of pulmonary vasodilator therapy. Patients with IPAH who are long-term vasoresponders exhibit significant reduction in mean PAP under calcium channel blockers (CCB) therapy and demonstrate the best survival outcomes among all pulmonary hypertension groups (1–4). O. Sitbon et al. (1) described acute changes in hemodynamics when using short-acting pulmonary vasodilators (intravenous administration of epoprostenol or nitric oxide inhalation) on a large population (*n* = 557) of patients with idiopathic PAH (IPAH), hereditary PAH (hPAH), drug- and toxin-associated PAH (dPAH) (1). The authors suggested a decrease in mean pulmonary artery pressure (mPAP) ≥ 10 mmHg with the achievement of mPAP ≤ 40 mmHg and an increase or the same level of cardiac output (CO) as criteria for a positive acute vasoreactive testing (AVT) (5). The selected criteria demonstrated a sensitivity of 69% and specificity of 87% in predicting positive clinical response to CCB therapy in patients with IPAH/hPAH/dPAH. Positive AVT was observed in 12.5% of IPAH patients (*n* = 70), but only 6.8% (*n* = 38) achieved long-term (more than 12 months) clinical response to high-dose CCB therapy, with a survival rate of 97% over 7 years of follow-up (3, 4).

A significant correlation was registered between the severity of pulmonary artery remodeling and a reduction in pulmonary artery pressure during AVT in IPAH patients (5–8). Isolated medial hypertrophy of the small pulmonary arteries was described in IPAH long-term responders to CCB (5). In long-term responders, mPAP reduction was achieved through pulmonary microvasculature recruitment and a pronounced decrease in pulmonary vascular resistance (PVR) (9). Near-complete normalization of lung perfusion in CCB responders was confirmed using PET CT data with 99mTc-PulmoBind, a substance that binds to the vascular endothelium adrenomedullin receptors (10). Medial hypertrophy, hyperplasia, and sclerosis of the intima of pulmonary arteris, plexiform lesion formation and thrombosis in situ observed in IPAH patients with negative AVT. Thus, a decrease in PVR and mPAP can be realized through the dilatation of larger pulmonary arteries (8). There are no non-invasive methods for *in vivo* assessment of pulmonary microvasculature morphology in PAH patients at present. Pulmonary vasodilation under powerful vasodilator used in AVT might indirectly reflect the severity of pulmonary vascular disease.

The Sitbon criteria for positive AVT reliably predicted short-term clinical response to CCB therapy in IPAH patients. However, nearly half of the AVT-positive patients experienced clinical deterioration and loss of vasoreactivity during the first 2 years of follow-up (3, 4, 11, 12). Patients with CCB therapy failure and vasoreactivity loss exhibited survival decline comparable to AVT-negative patients (4). Thus, the initial conventional criteria for positive AVT did not provide reliable long-term survival prognosis. The vast majority of IPAH patients did not meet the criteria for positive AVT. The magnitude of mPAP decrease during AVT was not taken into account in PAH severity evaluation or PAH-specific therapy choice. Risk stratification tools, including the 2022 ESC/ERS three-strata model and REVEAL 2.0, are generally applied to guide PAH-specific therapy and survival prediction in AVT-negative IPAH patients. High variability in PVR reduction was described in 181 PAH patients by Badagliacca et al. (13), even with combined PAH-specific therapy. One-third of patients experienced a 25% decrease in PVR from the baseline and failed to achieve low-risk status at 6 months of follow-up (13). Takano et al. (14) observed a positive correlation between the percentage of PVR decrease during AVT and improvement in symptoms, brain natriuretic peptide levels, and hemodynamics during 4-month therapy with pulmonary vasodilators in 48 patients with pulmonary hypertension associated with severe lung disease (14). The advent of new anti-remodeling therapies, and the existing limitations of PAH-specific vasodilators, has renewed interest in pulmonary vasoreactivity investigation.

We hypothesized that differences in pulmonary hemodynamics during AVT—described as pulmonary vasoreactive reserve (VRR) (15–17)—might be used to assess disease severity and prognosis in IPAH patients. This study aimed to evaluate the clinical significance of mPAP decrease during AVT in IPAH patients.

## Materials and methods

### Patient selection

Ninety adult (40.4 ± 14.6 years, 14.3% males) IPAH patients who underwent AVT were included from a prospective single-center PAH registry between 31 January 2007 and 1 June 2025 (18).

Exclusion criteria for the study were as follows: PAH etiologies other than IPAH, moderate to severe lung disease or diffusion capacity of lungs for carbon monoxide (DLCO) < 45%, left-heart disease (LHD), established malignancies, uncontrolled diabetes mellitus, uncontrolled thyroid disease, mental disorders, end-stage kidney dysfunction, and follow-up period of less than 6 months after diagnosis. The LHD phenotype was defined as having more than three comorbidities: systemic hypertension with two or more antihypertensive drugs, non-revascularized ischemic heart disease, left ventricular systolic or diastolic moderate-to-severe dysfunction defined by echocardiography, permanent atrial fibrillation, or obesity (body mass index (BMI) > 40 kg/m^2^) (Figure 1).

**Figure 1:**
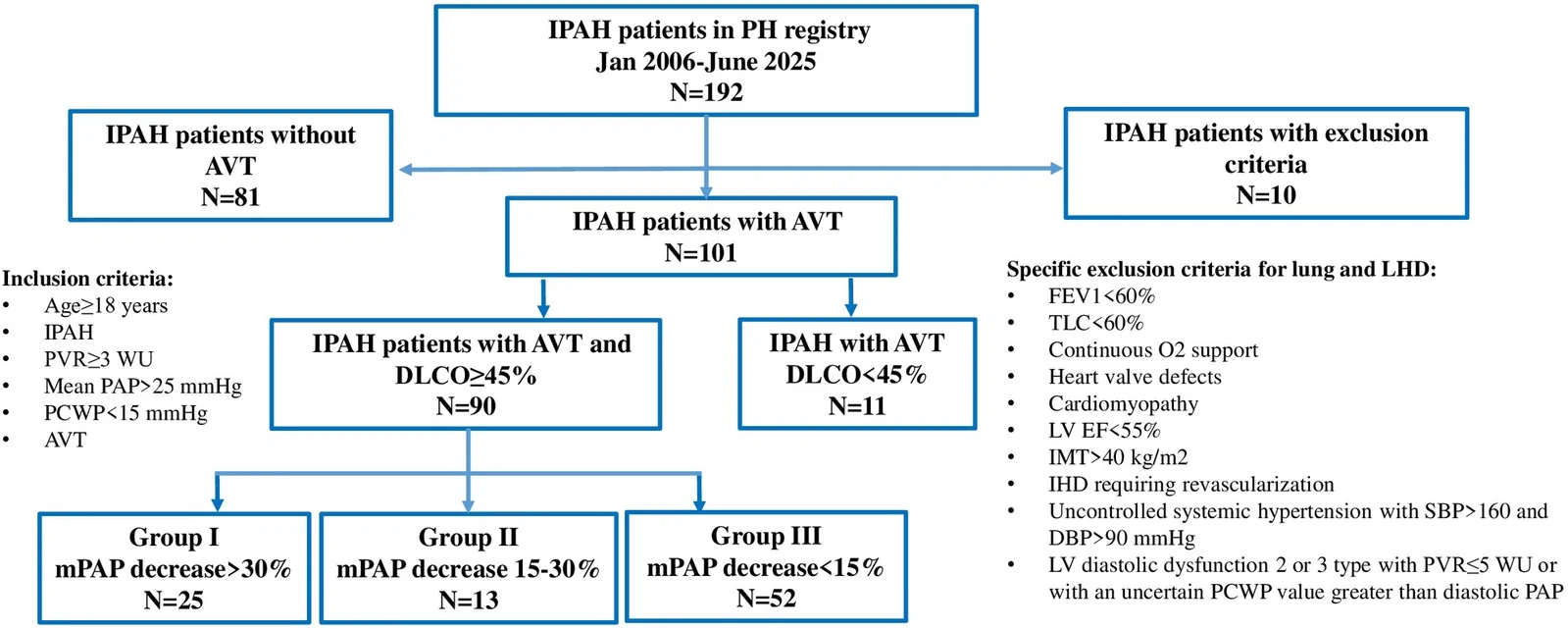
Study flowchart.

Ten prevalent IPAH patients were enrolled in the study without AVT performed at the regional center. These patients were reassessed at a reference center, including right-heart catheterization (RHC) with AVT, at 6.2 ± 2.4 months after initiation of PAH-specific therapy (sildenafil, endothelin receptor antagonist, or riociguat). Three of these prevalent patients demonstrated positive AVT. The effect of PAH-specific therapy on AVT results could not be reliably evaluated given the small number of patients.

Patients were divided into three groups based on the percent of mPAP decrease during AVT: Group I (*n* = 25): ΔmPAP > 30%; Group II (*n* = 13): ΔmPAP 15%–30%; and Group III (*n* = 52): ΔmPAP < 15%.

By legislation of the Russian Federation and the Local Acts of the Almazov National Medical Research Center, statistical analysis of registry data does not require specific permission from an ethics committee. The study reflects daily clinical practice and adheres to guidelines for the management of patients with PAH (19, 20). No identifiable patient information was presented in this study.

### Data collection

Demographic characteristics, symptoms, 6-min walk test (6MWT) distance, echocardiography, cardiac MRI, and cardiopulmonary exercise test (CPET) were collected at baseline within 1 month of RHC with AVT. Laboratory variables [hemoglobin, creatinine with estimated glomerular filtration rate (eGFR), and N-terminal pro-brain-type natriuretic peptide (NT-proBNP)] were collected at baseline within 1 month of RHC with AVT. Estimated GFR was calculated using the Chronic Kidney Disease Epidemiology Collaboration (CKD-EPI) equation.

Patients were characterized according to the initial three-strata ESC/ERS 2022 risk stratification approach (19) (https://pahriskcalc.github.io/3strata/calculator.html; URL accessed on December 1, 2025), classifying them as low-, intermediate-, and high-risk based on established values for the following parameters: symptoms (syncope and edema), WHO-functional class (FC), 6MWD, CPET (available in 69 patients), level of NT-proBNP, right-heart remodeling and RV systolic function (echocardiography and MRI in 53 patients), pericardial effusion, hemodynamics, and mixed venous oxygen saturation (SvO_2_).

The number of PAH medications and CCB prescription was assessed at the time of treatment initiation and at the end of follow-up.

### Right-heart catheterization and acute vasoreactive testing

RHC was performed and interpreted by experienced investigators. PAH was defined as mPAP ≥25 mmHg, pulmonary capillary wedge pressure (PCWP) <15 mmHg, and PVR >3 Wood units (WU), in accordance with national recommendations for pulmonary hypertension management (21). Patients with mPAP 20–24 mmHg and PVR <3 WU were not enrolled in to the study, as PAH therapy is not approved for these hemodynamic criteria. Another reason for the rejection was the unclear interpretation of AVT for mPAP 20–24 mmHg. Patients with PCWP greater than diastolic PAP were not included into the study. CO was measured by thermodilution or by the indirect Fick method in cases of patent foramen ovale. AVT was performed using inhaled iloprost (Bayer, Germany) 5–10 µg through a mouthpiece, with repeat hemodynamic measurements at 10–15 min (19–21). All hemodynamic measurements and blood gas analyses were collected before AVT and immediately after completion of iloprost inhalation. A positive AVT was defined as mPAP decrease ≥10 mm Hg from baseline, with mPAP ≤40 mmHg and no change or increase in cardiac output (19, 20). Total PVR was calculated using the following formula: ((mPAP-PAWP)/CO)*80, WU (19, 22). Pulmonary arterial compliance (PAC) was calculated as follows: stroke volume/(systolic pulmonary artery pressure − diastolic pulmonary artery pressure) (19).

We used three parameters to characterize the vasoreactive reserve: mPAP decrease (ΔmPAP), PVR decrease (ΔPVR), and PAC increase (ΔPAC) during AVT.

Parameters were presented as the absolute difference between baseline and iloprost inhalation values and the percentage of change from baseline. A ΔmPAP > 30% was defined as a high VRR, whereas ΔmPAP < 15% was defined as low VRR.

### MRI

Cardiac MRI was performed in 53 patients using a MAGNETOM Trio A Tim System 3 Tesla scanner (Siemens, Germany). Main pulmonary artery was measured for its relative area change and pulmonary artery distensibility (PAD) assessment (23). PAD was calculated using the following formula: PAD = [(maximum area − minimum area)/maximum area] × 100%. MRI was recorded within 30 days of the right-heart catheterization.

### CPET

CPET was performed in 69 patients using a cycle ergometer (Ebike, GI, USA) with an incremental workload of 10 W/min (RAMP-protocol)^1^ until symptoms-limited maximum tolerance. Gas exchange was measured by the breath-by-breath method using calibrated Oxycon Pro equipment (Cardinal Health, Germany).

### Follow-up of patients

The start date of follow-up was defined as the date of hospitalization for the first RHC with AVT. The last date of follow-up was the date of death, last patient visit, or telephone contact within 3 months. The dates and causes of death were obtained from medical records, local physicians, or death certificates provided by relatives. Follow-up assessments were captured every 3–6 months, determined by physicians based on the individual patient's healthcare needs. The primary outcome was all-cause mortality. The survival period was defined as the time between baseline RHC at a center and either death or the study end date of 25 December 2025. The number of PAH medications was assessed at the time of diagnosis and the end of follow-up.

All supporting data are available within the article and the Supplementary Material.

### Statistics

Demographic characteristics, PAH therapy, PAH FC (WHO), ESC/ERS three-strata risk status, smoking history, comorbidities, invasive hemodynamics, NT-proBNP, hemoglobin, eGFR, CPET and 6MWT results, and echocardiographic and MRI parameters were compared across the three VRR groups. Variables were tested for normality, and numerical parameters with a normal distribution were presented as mean ± standard deviation (M ± SD), while numerical parameters with an abnormal distribution were presented as median and interquartile range (IQR; M ± 25%; 75%). Categorical variables were presented as absolute numbers and percentages and compared using Fisher's exact test. The mean values of the three groups were compared using *t*-test or ANOVA, as appropriate. Pearson's correlation analysis was used to assess the direction and statistical strength between continuous variables. A statistically significant difference was determined as a two-tailed *p*-value less than 0.05. Factors associated with the ΔmPAP (%) during AVT were analyzed using general linear models.

The optimal cut-off value for % ΔmPAP during AVT was determined by maximizing Youden's J index derived from receiver operating characteristic (ROC) curve analysis, subject to statistical significance (*p* < 0.05) and hazard ratios significantly deviating from unity. Using SHapley Additive exPlanations (SHAP) analysis of the naive model, we assessed the contribution of % ΔmPAP to the predicted mortality risk in relation to absolute mPAP values. A significant point of inflection was identified at approximately 6% on the distribution curve of % ΔmPAP. This meant that a % ΔmPAP greater than 6% was associated with lower mPAP values in AVT and a lower probability of patient mortality (negative values on the SHAP value scale) (Supplementary Figure S2A). ROC curve analysis and Youden's J calculation for the naive model confirmed that the point of inflection occurred at 6%. However, a plateau was reached at 15%, and this value was chosen as the threshold for splitting in advanced LightGBM models (Supplementary Figure S2B).

Survival was assessed using Kaplan–Meier curves over 14 years. Predictors of mortality were evaluated using univariate Cox proportional hazards models in the entire cohort of IPAH patients. Selected variables with *p* < 0.05 and hazard ratio different from 1 were analyzed using the multivariate Cox proportional hazards model.

Data were analyzed using Statistica for Windows, version 10.0 (StatSoft, Tulsa, OK, USA). Missing variables were handled using listwise deletion (complete-case analysis); no imputation (e.g., mean substitution) was performed.

### Mathematical experimental modeling

We compared the standard model of survival prediction using the 2022 ESC/ERS three-strata risk stratification scale and the experimental model of risk stratification. The experimental model was based on the 2022 ESC/ERS three-strata scale with the inclusion of the degree of Δ%mPAP during AVT. In the model, ΔmPAP >30% was classified as a low-risk category, ΔmPAP 15%–30% as intermediate risk, and ΔmPAP <15% as a high-risk category.

The anonymized dataset was initially partitioned into training and test using in a 50/50 ratio. This non-standard split was motivated by the limited size of the dataset. A fixed random seed was used throughout all stages of the study (random seed = 529894210) to ensure reproducibility.

For hyperparameter optimization during model training, the training set was further subdivided into training and validation subsets following a k-fold cross-validation scheme (three-fold). The optimal hyperparameter configuration was subsequently used to train the model on the entire training set. The trained model was then applied once to the test set to predict the 2022 ESC/ERS three-strata risk score factor. The following metrics were recorded: MAPE (Mean Absolute Percentage Error), C-index (Concordance Index), and raw predicted ESC scores.

The experiment was repeated with inclusion of the experimental feature Δ%mPAP in AVT. A paired bootstrap test of superiority probability was performed on the raw predicted 2022 ESC/ERS three-strata scores to assess differences between the two model variants.

## Results

### Vasoreactive reserve characteristics

The distribution of mPAP and PVR decrease, PAC increase in AVT presented in absolute values and the percent from baseline in entire IPAH population” on “The distribution of hemodynamic responce to AVT illustrated on Figure 2.

**Figure 2:**
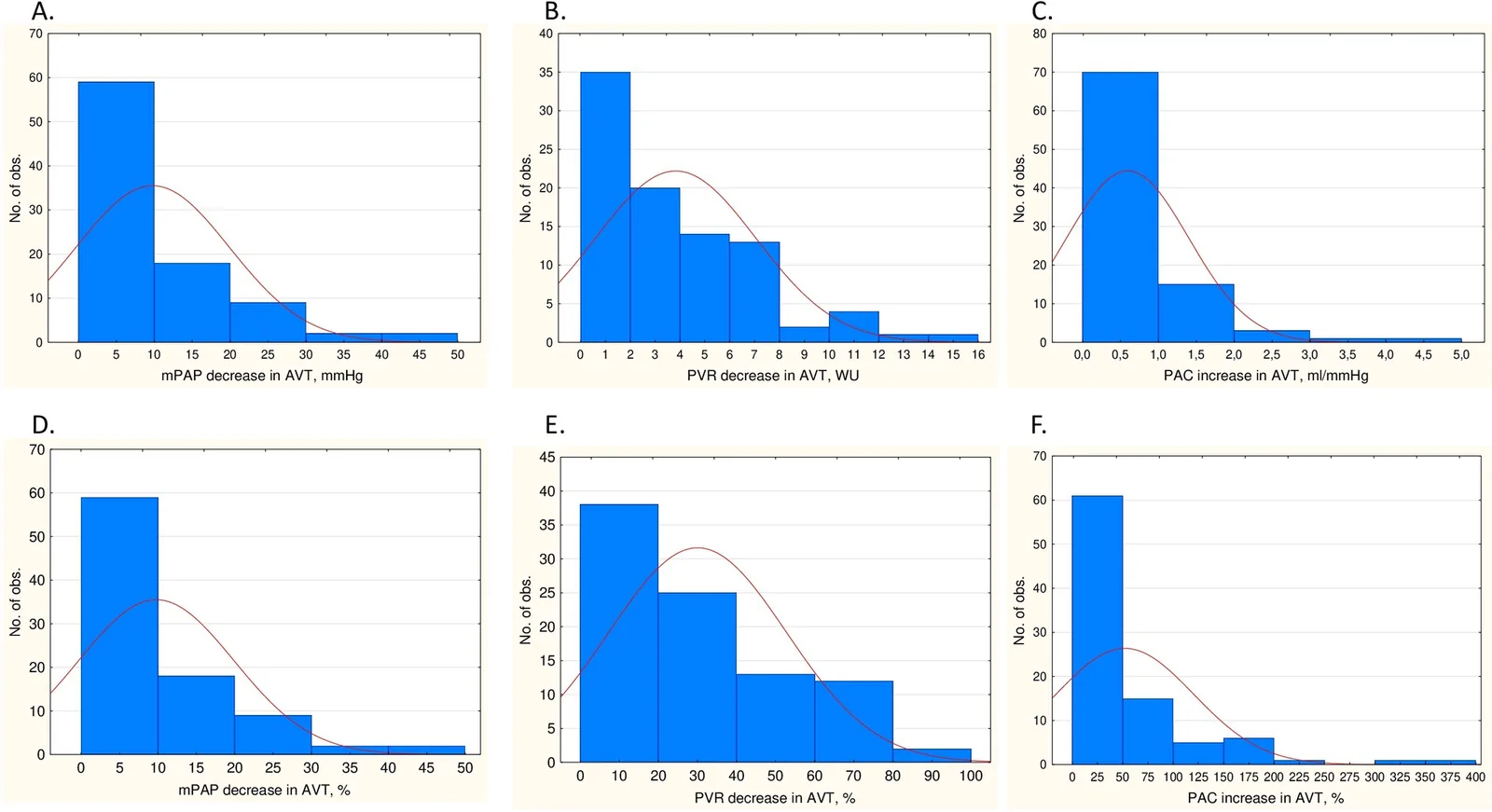
The distribution of mPAP, PVR decrease in AVT, and PAC increase in AVT in absolute values **(A–C)** and percent of changes from the baseline in AVT **(D–F)**. AVT, acute vasoreactive testing; mPAP, mean pulmonary arterial pressure; PAC, pulmonary artery compliance; PVR, pulmonary vascular resistance.

A mean ΔPAP <15% was most frequently observed (57%). Patients with ΔmPAP >30% accounted for 27.7%, while 14.4% of IPAH patients demonstrated ΔmPAP between 15% and 30%. The absolute value of ΔmPAP during AVT was 21 mmHg (14; 28) in Group I, 11 mmHg (10; 12) in Group II, and only 2.5 mm Hg (1; 4.5) in Group III. Ninety-two percent of patients in Group I and 30.7% in Group II fulfilled Sitbon criteria for positive AVT.

The ΔPVR was 3.0 WU (1.4; 5.9) in entire cohort, which corresponded to 27% ΔPVR from the baseline (11.1; 47.0). The majority of patients (41.1%) exhibited ΔPVR > 30% (*n* = 37), while ΔPVR < 15% was registered in 34.4% (*n* = 31) and ΔPVR 15%–30% in 23.3% (*n* = 21) patients. In Group I, all patients demonstrated ΔPVR > 30%, while 100% of Group III patients exhibited ΔPVR < 15%. Different values of ΔPVR were noted in Group II: >30% in 46.1% patients, 15%–30% in 38.5%, and ΔPVR < 15% in 15.4%.

No differences in demography, comorbidities, or prevalence status were registered in the three groups of ΔmPAP (Supplementary Table S1, 1.1). Lower body mass index (BMI) was noted for Group I compared to Groups II and III. Patients with BMI >30 kg/m^2^ were older compared to patients with BMI <30 kg/m^2^ (46.5 ± 12.7 vs. 37.5 ± 14.7 years, *p* = 0.009). Patients with BMI >30 kg/m^2^ did not differ in mean blood pressure (*p* = 0.7), PCWP (*p* = 0.3), mPAP (*p* = 0.7), and PVR (*p* = 0.2) compared with patients with BMI < 30 kg/m^2^.

Higher FC PAH (WHO), severely impaired physical performance in 6MWT and CPET, worse hemodynamic profile [higher heart rate (HR), right atrial pressure, PVR, mPAP and lower mean blood pressure, cardiac index (CI)], right-heart dilatation, and RV dysfunction were observed in Group III compared with Group I patients (Supplementary Table S1, 1.1). The majority of the evaluated parameters did not differ significantly between Group II and Group III patients.

Baseline PAC and PAC during AVT were quite low across the entire cohort [0.95 (0.65; 1.3) mL/mmHg and 1.32 (0.82; 2.4) mL/mmHg, respectively]. Group I significantly differed in baseline PAC, PAC during AVT, and ΔPAC from Group III. No differences in baseline PAC and PAC during AVT were registered between Groups II and III, whereas absolute value and the percent of ΔPAC from the baseline were markedly lower in Group III compared with Group II and Group I (Table 1, Figure 3).

**Table 1:** Characteristics of IPAH patients depending on percent of mean pulmonary arterial pressure decrease in acute vasoreactive testing.

Parameters, *n* (%); M ± SD; Me (IQR 25%; 75%)	All patients, *n* = 90	Group I mPAP _decrease_ > 30% *n* = 25	Group II mPAP _decrease_ 15–30% *n* = 13	Group III mPAP _decrease_ < 15% *n* = 52	*p*
Age, years	41.2 ± 14.4	37.2 ± 14.6	44.2 ± 15.1	42.4 ± 14.0	0.2
Male*, *n* (%)	12 (13.3)	3 (12)	2 (15.4)	7 (13.5)	NA
Prevalent, *n* (%)	10 (11.1)	3 (12)	1 (7.7)	6 (11.5)	NA
AVT+, *n* (%)	27 (30)	23 (92)	4 (30.7)	0	NA
6MWT, m	360.9 ± 121	425.5 ± 118	324.2 ± 124.2	339.1 ± 113	0.02^a^; 0.7^b^; 0.003^c^
III FC PAH*, *n* (%)	61 (67.7)	10 (40)	11 (84.6)	40 (76.9)	0.02
BMI, kg/m^2^	27.2 ± 6.4	23.9 ± 4.2	28.1 ± 5.0	28.5 ± 7.1	0.01^a^; 0.8^b^; 0.004^c^
Smoking, *n* (%)	10 (11.1)	3 (12)	2 (15.4)	5 (9.6)	Na
Laboratory					
NT-proBNP, pg/mL	805 [385; 2,091]	385 [125; 811]	1,100 [739; 1,883]	1,430 [574; 2,456]	0.5^a^; 0.9^b^; 0.3^c^
Creatinine, µmol/L	82.5 ± 20.4	76.5 ± 23.2	86.5 ± 15.2	84.4 ± 19.8	0.2
eGFR, mL/min/1.73 m^2^	90.5 ± 26.5	93.7 ± 24.1	88.9 ± 20.6	89.3 ± 28.8	0.8
Hemoglobin, g/L	145.7 ± 19.3	138.5 ± 18.7	151.6 ± 19.1	147.8 ± 19.0	0.048^a^; 0.5^b^; 0.046^c^
Hemodynamics					
mPAP, mmHg	56.9 ± 15.2	51.5 ± 13.1	55.1 ± 10.6	60.1 ± 16.5	0.4^a^; 0.3^b^; 0.02^c^
mPAP_AVT, mmHg	47.3 ± 18.5	28.7 ± 8.5	43.9 ± 10.5	57.0 ± 16.4	0.00002^a^; 0.008^b^; <0.00001^c^
ΔmPAP, mmHg	6.5 [2; 14]	21 [14; 28]	11 [10; 12]	2.5 [1; 4.5]	0.0002^a^; <0.00001^b^^;^c^^
ΔmPAP,%	11.6 [3.1; 31.4]	43.2 [32.5; 48.3]	19.0 [16.3; 26.2]	3.8 [2.2; 8.1]	<0.00001^a^^,^b^,^c^^
RAP, mmHg	7.1 ± 4.8	4.9 ± 3.3	8.3 ± 6.6	7.7 ± 4.7	0.04^a^; 0.7^b^; 0.008^c^
PAWP, mmHg	7.6 ± 3.8	7.4 ± 3.8	6.5 ± 4.1	8.5 ± 3.8	0.5^a^; 0.2^b^; 0.5^c^
CI, L/min/m^2^	2.27 ± 0.7	2.72 ± 0.7	2.2 ± 0.5	2.1 ± 0.7	0.047^a^; 0.4^b^; 0.001^c^
PVR, WU	12 [8.4; 17.8]	9.7 [6.5; 12.0]	11.0 [9.4; 14.8]	15.3 [9.3; 19.7]	0.3^a^; 0.1^b^; 0.004^c^
PVR_AVT, WU	8.2 [4.5; 14.5]	3.2 [2.5; 4.6]	8.0 [6.5; 10.3]	12.7 [7.7; 17.8]	0.00005^a^; 0.03^b^; <0.00001^c^
ΔPVR, WU	3.0 [1.4; 5.9]	5.4 [3.5; 7.3]	3.3 [2.1; 4.7]	1.8 [1.0; 3.9]	0.01^a^; 0.5^b^; 0.0002^c^
ΔPVR, %	27 [11.1; 47]	62.7 [52.2; 67.3]	29.8 [18.5; 37.6]	12.8 [6.8; 25.9]	<0.00001^a^; 0.005^b^; <0.00001^c^
SatO_2_, %	96 [93; 97]	97 [96; 98]	95 [90; 97]	94 [92; 96]	0.004^a^; 0.8^b^; 0.0002^c^
SvO_2_, %	62.4 ± 10.2	69.1 ± 6.9	59.9 ± 14.4	59.8 ± 9.1	0.01^a^; 0.9^b^; <0.0001^c^
PAC, mL/mm Hg	0.95 [0.65; 1.3]	1.17 [0.9; 2.0]	0.83 [0.8; 1.2]	0.8 [0.57; 1.2]	0.1^a^; 0.5^b^;0.002^c^
PAC_AVT, mL/mmHg	1.32 [0.82; 2.4]	2.86 [2.35; 3.66]	1.07 [0.97; 1.66]	0.9 [0.64; 1.47]	0.0004^a^; 0.1^b^; <0.0,00,001^c^
ΔPAC, mL/mmHg	0.23 [0.08; 0.74]	1.57 [1.05; 1.86]	0.23 [0.19; 0.59]	0.11 [0.05; 0.25]	0.00008^a^; 0.001^b^; <0.0,00,001^c^
ΔPAC,%	26.9 [11.2; 70.3]	106 [72; 180]	27 [25; 34]	16.2 [6; 27.6]	0.0005^a^; 0.01^b^;<0.00001^c^
Echocardiography					
LAVI, mL/m^2^	25.5 ± 7.7	25.1 ± 4.6	26.2 ± 7.0	24.4 ± 9.1	0.6^a^; 0.8^b^;0.9^c^
EDD LV, mm	41.5 ± 11.2	42.4 ± 4.9	42.1 ± 4.9	40.8 ± 14.2	0.8^a^;0.7^b^; 0.6^c^
Lateral E/e’ ratio	6.4 ± 0.2	5.9 ± 0.4	5.9 ± 0.5	6.8 ± 2.4	0.9^a^; 0.3^b^; 0.1^c^
RA area, cm^2^	26.0 [21.0; 34.0]	20 [18.5; 24.0]	27.5 [23.0; 30.0]	28.0 [24.0; 37.0]	0.03^a^, 0.3^b^; 0.0002^c^
RV base. A4С, mm	46.8 ± 6.4	44.2 ± 7.2	46.0 ± 4.9	48.3 ± 5.9	0.4^a^; 0.2^b^; 0.01^c^
EDD RV/EDD LV	1.2 ± 0.3	1.0 ± 0.2	1.1 ± 0.2	1.3 ± 0.3	0.5^a^; 0.02^b^; 0.0001^c^
IVC, mm	20 [17; 24]	18 [16; 22]	22 [17; 25]	21 [18; 25]	0.05^a^; 0.8^b^; 0.01^c^
TAVs, cm/s	9 [8; 11]	10 [9; 12]	9 [8; 10]	9 [7; 10]	0.1^a^; 0.5^b^; 0.009^c^
TAPSE/SPAP, mm/mmHg	0.18 [0.13; 0.28]	0.20 [0.18; 0.31]	0,18 [0.15; 0.29]	0.16 [0.12; 0.22]	0.3^a^; 0.2^b^; 0.003^c^
MRI, *n* = 53 (18/7/28)					
RA short dimension, mm	51.9 ± 10.8	46.8 ± 8.6	52.6 ± 10.8	54.9 ± 11.3	0.2^a^; 0.6^b^;0.02^c^
RVESV index, mL/m^2^	57.1 [44.2; 69.4]	47.9 [33.3; 61.8]	60.1 [51.8; 68.9]	62.3 [48.8; 78.4]	0.3^a^; 0.4^b^; 0.02^c^
RVEF, %	34.4 ± 10.8	38.0 ± 11.1	34.7 ± 8.6	31.4 ± 10.8	0.5^a^; 0.5^b^; 0.05^c^
LV SV index, mL/m^2^	34.8 ± 11.8	36.7 ± 10.7	39.1 ± 14.7	32.4 ± 11.6	0.6^a^; 0.2^b^; 0.2^c^
LVEF, %	60.1 ± 10.4	60.7 ± 6.7	61.7 ± 8.5	59.5 ± 12.7	0.7^a^; 0.6^b^; 0.7^c^
RV ESVi/LV ESVi	2.6 [1.7; 3.6]	1.8 [1.3; 2.6]	2.8 [2.3; 3.2]	3.4 [2.3; 4.5]	0.4^a^; 0.3^b^; 0.03^c^
PAD, %	8.05 [4.6; 17.3]	17.8 [8.3; 25.8]	11.6 [4.1; 18.6]	5.9 [4.0; 10.6]	0.1^a^; 0.2^b^; 0.0001^c^
CPET, *n* = 69					
VO_2_ peak, mL/min/kg	12.2 [10.2; 17.3]	17.3 [11.1; 22]	12.2 [9.1; 15.2]	11.2 [9.8; 13.8]	0.04^a^; 0.4^b^; 0.0001^c^
VO_2_ peak Predicted, %	53.3 ± 17.2	63 ± 17.4	55.8 ± 10.5	44.3 ± 15.9	0.2^a^; 0.1^b^; 0.0005^c^
V′O_2_/HR Predicted, %	62.8 ± 18.1	70.0 ± 18.3	66.1 ± 14.9	57.9 ± 17.5	0.6^a^; 0.2^b^; 0.01^c^
VE/VCO_2_	50.3 ± 12.6	40.6 ± 9.1	51.9 ± 11.9	55.3 ± 11.6	0.007^a^; 0.4^b^; 0.000002^c^
ESC/ERS 2015 risk status and mortality					
Score	2.03 ± 0.5	1.68 ± 0.4	2.06 ± 0.4	2.18 ± 0.4	0.02^a^; 0.3^b^; 0.00001^c^
Low risk, *n* (%)	16 (17.7)	10 (40)	1 (7.7)	5 (9.6)	0.004 (*χ*^2^ = 15.2)
Intermediate risk, *n* (%)	56 (62.2)	13 (52)	11 (84.6)	32 (61.5)	
High risk, *n* (%)	18 (20)	2 (8)	1 (7.7)	15 (28.8)	
Death*, *n* (%)	17 (18.8)	0	2 (15.4)	15 (28.8)	0.001 (χ^2^ = 9.3)

6MWT, 6-minute walk test; AVT, acute vasoreactive testing; BMI, body mass index; CCB, calcium channel blockers; CI, cardiac index; EDD LV, end-diastolic dimension of left ventricle; EDD RV/EDD LV, the ratio of the end-diastolic dimension of the right ventricle to the end-diastolic dimension of the left ventricle; lateral E/e’ratio, the ratio of early diastolic mitral inflow velocity to early diastolic lateral mitral annulus velocity; eGFR, estimated glomerular filtration rate (the estimated GFR was calculated according to the CKD-EPI equation); ePAP, estimated pulmonary artery pressure; ESVi, end systolic volume index; FC, functional class; IPAH, idiopathic pulmonary arterial hypertension; IVC, inferior vena cava; IQR, interquartile range; LAVI, left atrium volume index; LVEF, left ventricular ejection fraction; mPAP, mean pulmonary artery pressure; NT-proBNP, N-terminal fragment of B-type brain natriuretic peptide; PAC, pulmonary artery compliance; PAD, pulmonary artery distensibility; PAH, pulmonary arterial hypertension; PCWP, pulmonary capillary wedge pressure; PET CO_2_, end-tidal carbon dioxide partial pressure; PVR, pulmonary vascular resistance; RA, right atrial; RAP, right atrial pressure; RHC, right-heart catheterization; RV, right ventricle; SatO_2_, arterial blood saturation; SV, stroke volume; SVi, stroke volume index; SvO_2_, mixed venous blood saturation; TAPSE, tricuspid annulus plane systolic excursion; TAVs, systolic tricuspid annular velocity; VE/VCO_2_, the ratio of minute ventilation to carbon dioxide production; VO_2_ peak, peak oxygen consumption; V′O_2_/HR, oxygen pulse. Δ-difference in values in acute vasoreactive testing.

a *p*-value between group I and II.

b *p*-value between group II and III.

c *p*-value between group I and III.

**Figure 3:**
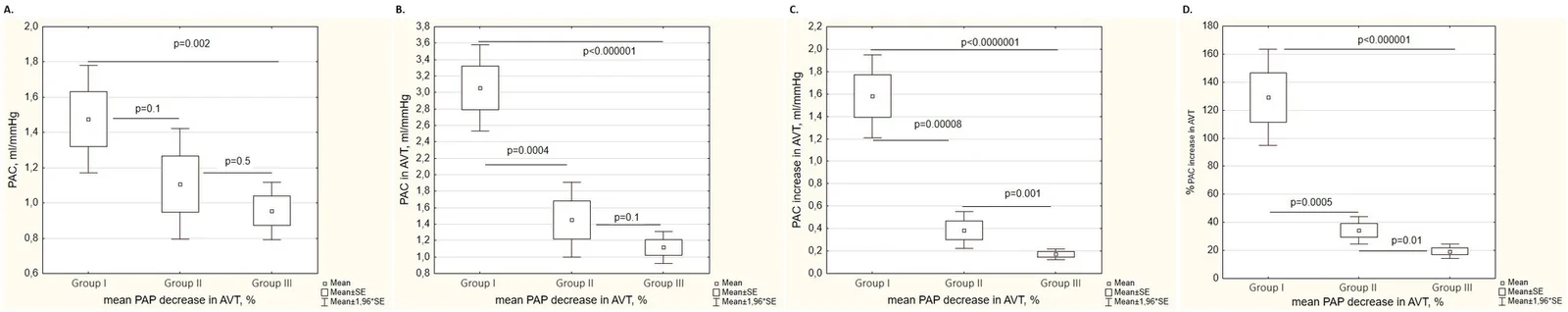
Pulmonary artery compliance at baseline **(A)**, in AVT **(B)**, PAC increase in AVT in absolute values **(C)**, and % PAC increase in AVT from baseline **(D)** in three VRR groups.

Pulmonary artery distensibility significantly differed between Groups I and III (*p* = 0.0001). No differences in PAD between Groups I and II or Groups II and III were registered (Figure 4), likely explained by the low number of patients in Group II.

**Figure 4:**
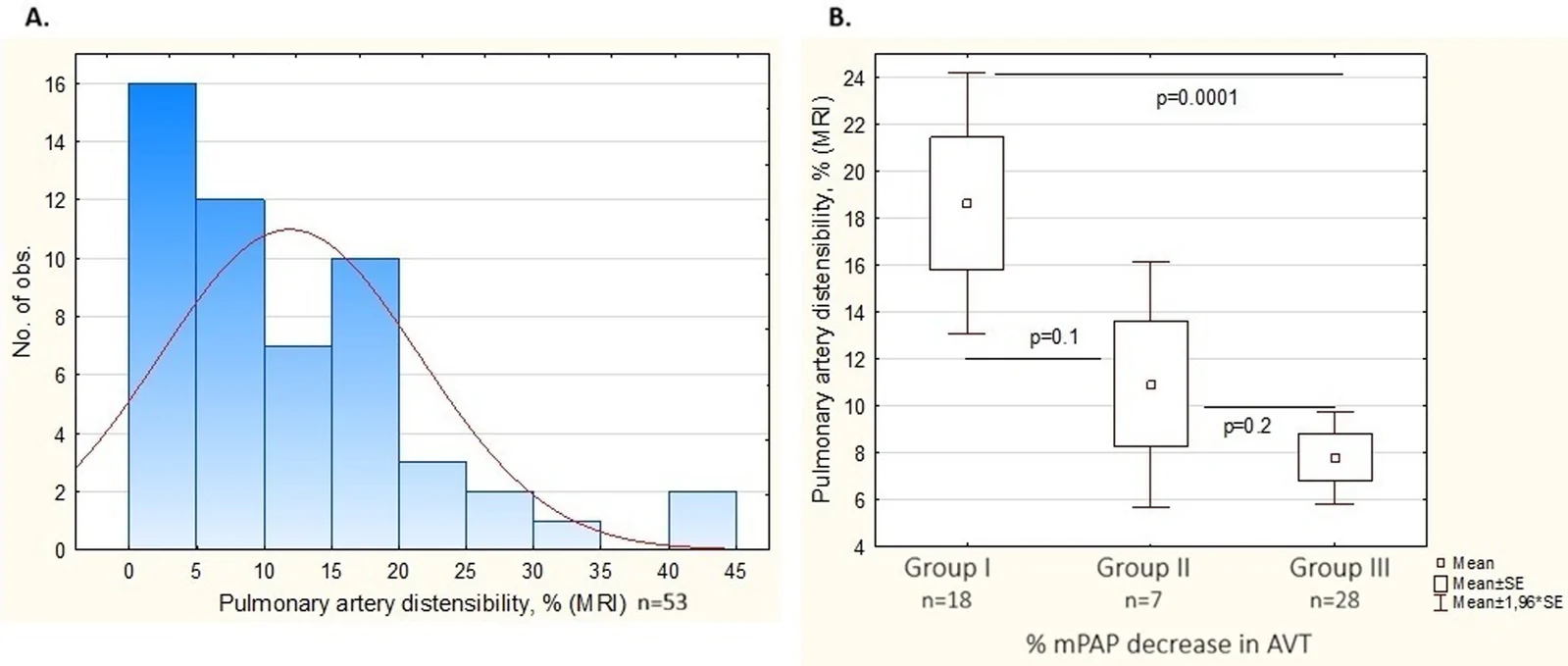
Pulmonary artery distensibility (MRI) distribution **(A)**, and pulmonary artery distensibility depending on the mean PAP decrease **(B)**.

### Factors associated with pulmonary vascular reserve

Syncope presence was associated with higher Δ%mPAP (r = 0.24, *p* = 0.02), NT-proBNP concentration in serum (*r* = − 0.26, *p* = 0.001), Significant association was revealed between ∆%mPAP and SatO_2_ (*r* = 0.42, *p* = 0.00008), serum creatinine (*p* = 0.028), hemoglobin (*p* = 0.0027), C-reactive protein (*r* = −0.26, *p* = 0.01), right atrial square (*r* = − 0,41, *p* = 0,00,006), the ratio of the end-diastolic dimension of the right ventricle to the end-diastolic dimension of the left ventricle (RVEDD/LVEDD) (*r* = − 0.41, *p* = 0.00001), right ventricular end systolic volume index (RVESV index) (*r* = − 0.34, *p* = 0.02), systolic tricuspid annular velocity (TAVs) (*r* = 0.37, *p* = 0.0004), tricuspid annulus plane systolic excursion (TAPSE)/systolic pulmonary arterial pressure (SPAP) (*r* = 0.29, *p* = 0.01), peak oxygen consumption (VO_2_ peak) (*r* = − 0.56, *p* < 0.00001), VO_2_ peak % Predicted (*r* = 0.53, *p* < 0.00001), oxygen pulse (V′O_2_/HR) % Predicted (*r* = 0.45, *p* = 0.001), the ratio of minute ventilation to carbon dioxide production (VE/VCO_2_) (*r* = − 0.56, *p* < 0.00001) (Figure 5), end-tidal carbon dioxide partial pressure (PET CO_2_) at rest and peak (*r* = 0.36, *p* = 0.004; *r* = 0.52; *p* = 0.00004, respectively), right atrial pressure (*r* = − 0.26, *p* = 0.01), PAC (*r* = 0.30; *p* = 0.0045), and PAD (*r* = 0.37, *p* = 0.0058) (Figure 6). We did not register any significant differences in baseline and AVT hemodynamics, echocardiography, MRI parameters, NT-proBNP level, 6MWT, or CPET results between incident and prevalent patients.

**Figure 5:**
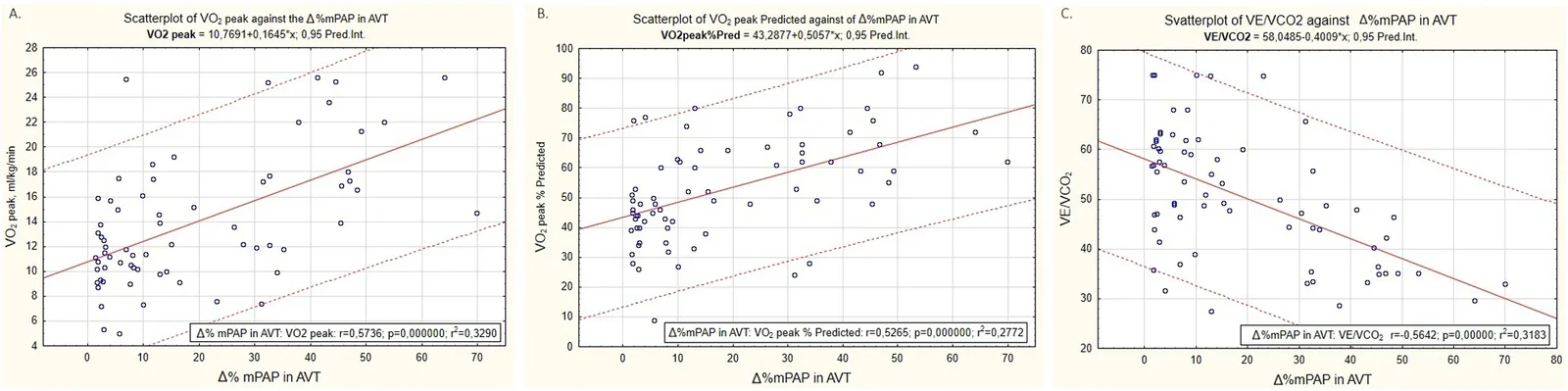
Correlation between the percent of ΔmPAP in acute vasoreactive testing and peak oxygen consumption **(A)**, the percent of predicted peak oxygen consumption **(B)**, and the ratio of minute ventilation to carbon dioxide production **(C)**.

**Figure 6:**
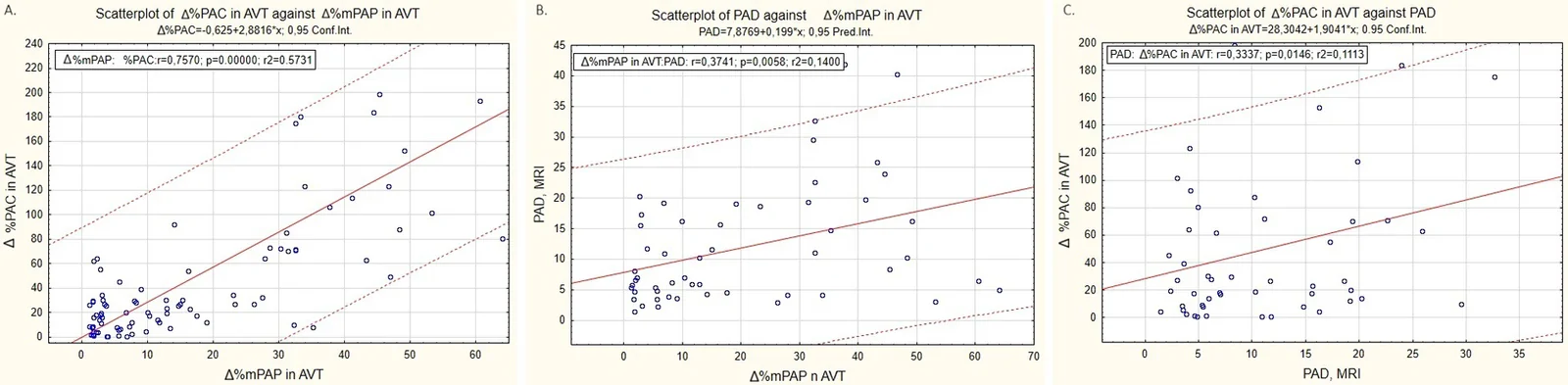
Correlation between the mean Δ%PAP in acute vasoreactive testing and the percent of pulmonary artery compliance increase in AVT **(A)**, pulmonary artery distensibility **(B)**, and Δ%PAC and pulmonary artery distensibility **(C)**.

No significant correlations were registered between DLCO and mPAP or PVR at a baseline during AVT. However, direct correlations between DLCO and baseline PAC (*r* = 0.21, *t* = 2.06, *p* = 0.04), PAC during AVT (*r* = 0.23, *t* = 2.26, *p* = 0.03), and ΔPAC (*r* = 0.24, *t* = 2.3, *p* = 0.02) were registered.

### Factors related to death

Seventeen (18.8%) patients died across the cohort during a follow-up of 2.32 years (1.04; 4.06). All deaths were due to PAH-related causes. The survival rate was 93%, 93%, 83%, 78%, 62%, and 55% at 1, 2, 3, 5, 7, and 10 years, respectively (Figure 7A). Significant difference in survival was registered between Group I and Group II (*p* = 0.049), and Group I and Group III (*p* = 0.007). However, no differences were observed in patients from Groups II and III (Figure 7B). No deaths were registered in Group I. Almost all deaths (15/17, 88.2%) were registered in Group III, with the lowest Δ%mPAP.

**Figure 7:**
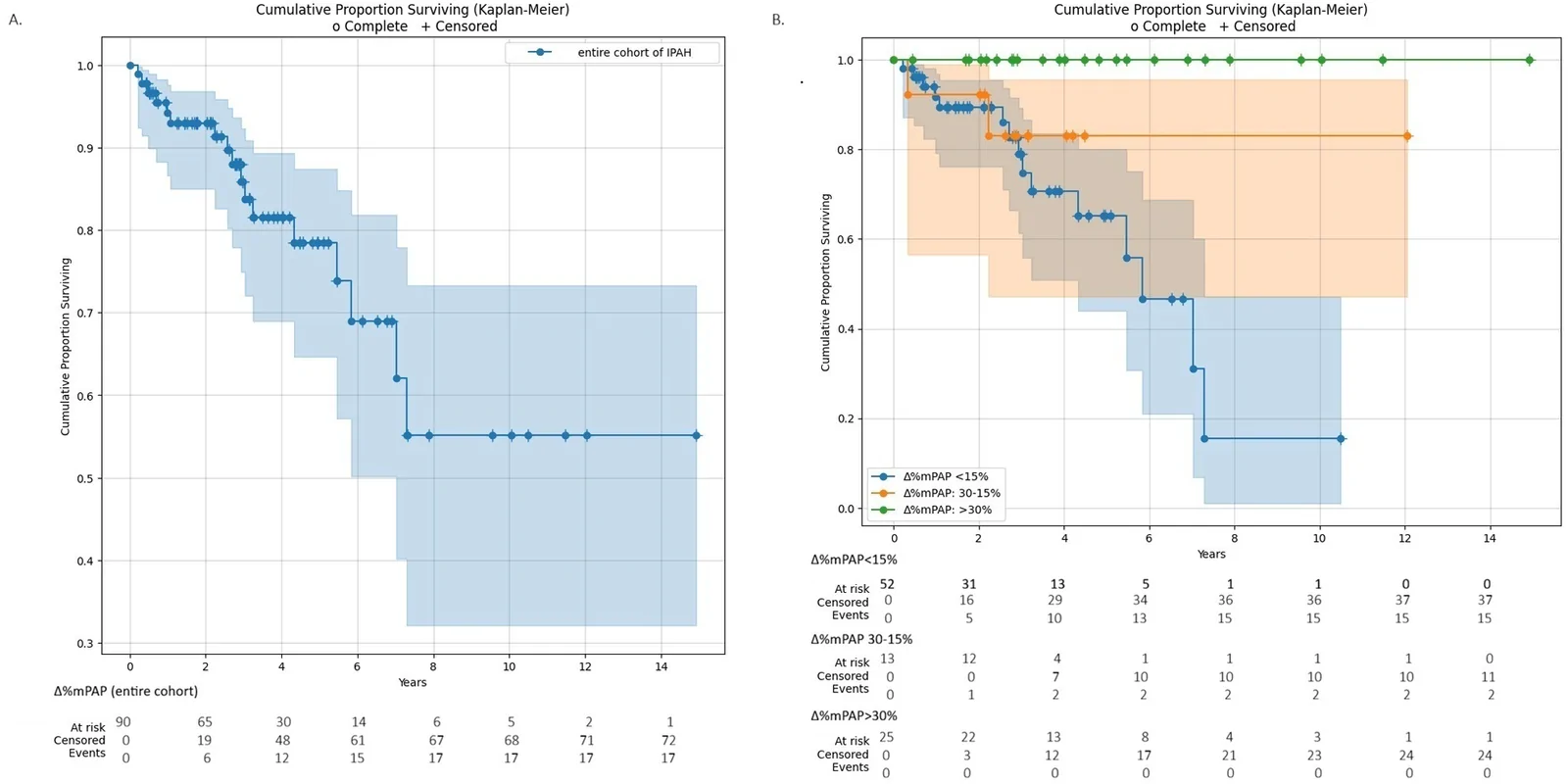
Survival of IPAH patients: in entire cohort **(A)** and depending on percent of mean pulmonary artery pressure decrease in acute vasoreactive testing in patients with IPAH **(B)**.

No difference in baseline mPAP was registered between living and deceased patients (56.4 ± 14.5 vs. 59.6 ± 17.9 mmHg, *p* = 0.4), whereas mPAP during AVT was markedly lower in living patients (45.4 ± 17.9 vs. 55.3 ± 19.3 mmHg, *p* = 0.04). The majority of deceased patients had baseline mPAP values >40 mmHg (82.3%). The absolute value of ∆mPAP and the percent from the baseline was higher in alive patients compared to dead [7 mmHg (2; 16) vs. 3 (2; 7), *p* = 0.01] and [14.1% (3.8; 32.5) vs. 5.6 (3; 11.7), *p* = 0.01].

Living and deceased patients did not differ in baseline PVR [11 (7.8; 17.2) vs. 15.9 (10.1; 20.1) WU, *p* = 0.06] and the absolute value of ΔPVR during AVT [3.1 (1.47; 6.1) vs. 2.2 (1.1; 4.2) WU, *p* = 0.3]. The percent of ΔPVR was significantly higher in living patients than deceased patients [28% (12.4%; 52.2%) vs. 15.9% (8.7%; 27.9%)%, *p* = 0.04].

No significant difference in baseline PAC [0.97 (0.73; 1.35) vs. 0.79 (0.59; 1.04) mL/mmHg, *p* = 0.07] was revealed between living and deceased patients. However, during acute vasoreactive testing, PAC was significantly higher in living patients compared to deceased patients [1.5 (0.9; 2.72) vs. 0.9 (0.6; 1.4) mL/mmHg, *p* = 0.01]. Absolute PAC value was markedly elevated in living patients [0.29 (0.13; 1.1) vs. 0.14 (0.07; 0.23) ml/mm Hg, *p* = 0.02], but the percent of ΔPAC did not reach statistical significance in living patients compared with those deceased [27% (12%; 72.6%) vs. 26.2% (8.9%; 30%), *p* = 0.053).

Deceased patients had a lower cardiac index at a baseline [1.7 (1.5; 2.17) vs. 2.26 (1.8; 2.8) L/min/m^2^, *p* = 0.02] and lower DLCO compared with living patients (58.9% ± 13.5% vs. 73.7% ± 14.3%, *p* = 0.0002).

Significant differences were observed in survival, depending on the 2022 ESC/ERS three-strata risk stratification (Figure 8).

**Figure 8:**
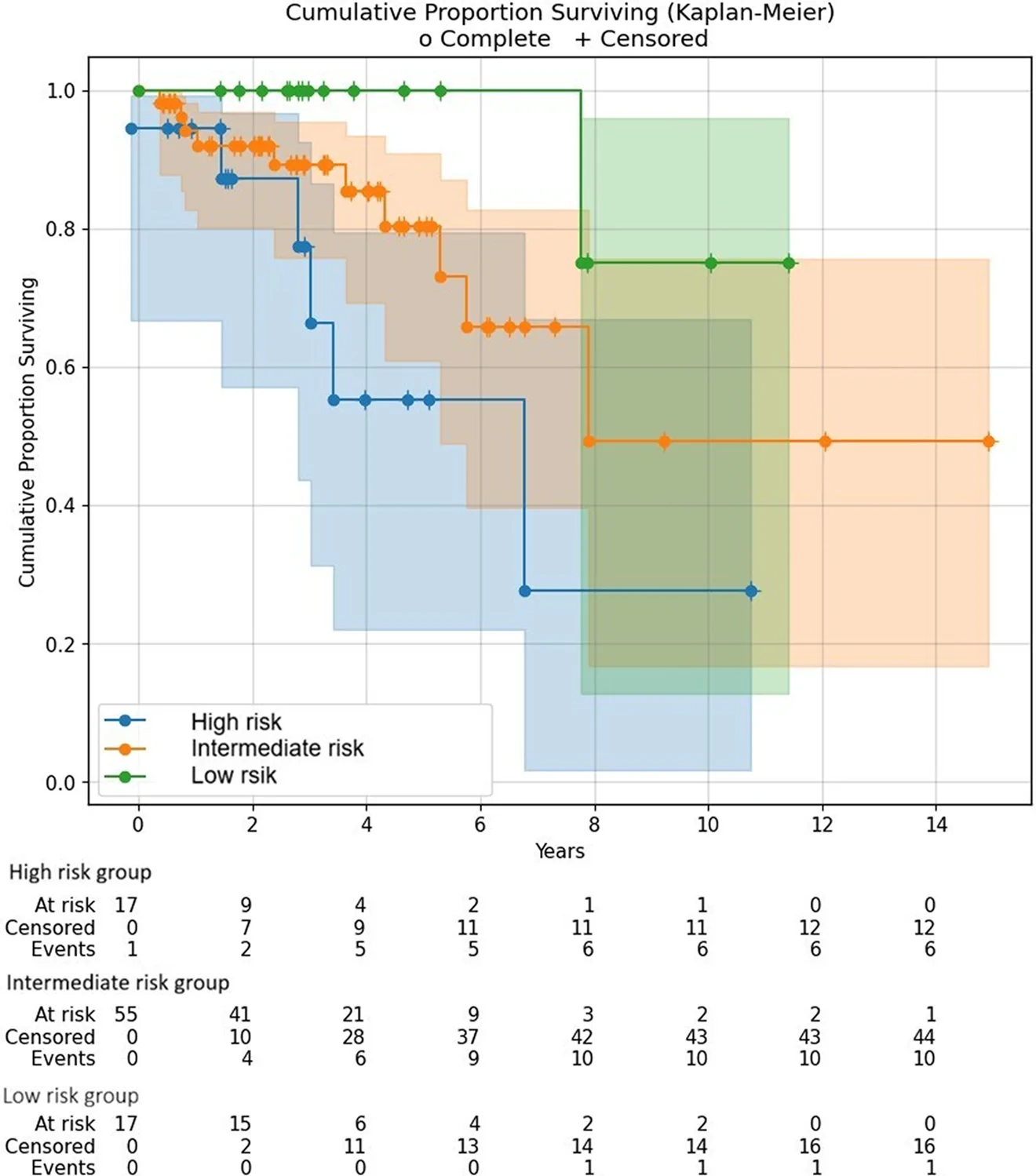
Survival of IPAH patients depending on the 2022 ESC/ERS three-strata risk classification.

Baseline mPAP differed significantly between low-, intermediate-, and high-risk 2022 ESC/ERS three-strata categories of IPAH patients (43.8 ± 9.9 vs. 57.6 ± 14.5 vs. 66.8 ± 13.3 mmHg, *p* = 0.00002). Significant differences in percent of ΔmPAP during AVT were registered in the three risk groups [low risk: 32.4% (12.3; 42.2) vs. intermediate risk: 12.9% (3.01; 28.2) vs. high risk: 4.8% (1.8; 8.9), *p* = 0.0009] (Figure 9). Differences in absolute values of ΔmPAP did not reach statistical significance among the risk categories [12.5 (5.0; 18.0) vs. 6.5 (2.0; 13.0) vs. 3.0 (1.0; 7.0) mmHg, *p* = 0.07, respectively].

**Figure 9:**
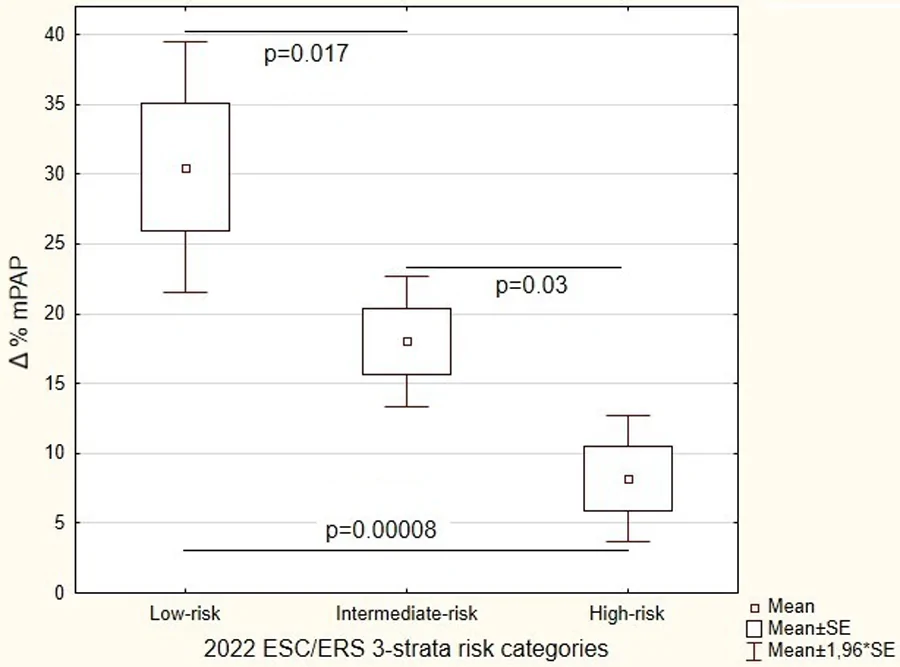
The percent of mean pulmonary artery pressure decrease depending on the 2022 ESC/ERS three-strata risk stratification categories in 90 IPAH patients.

### Therapy

CCBs and PAH-specific therapies—including sildenafil, riociquate, bosentan, macitentan, ambrisentan, selexipag, and inhaled iloprost—were administered to patients. Parenteral prostanoids were unavailable in the country.

### Initial therapy

CCB therapy was prescribed for 18 patients, PAH-specific monotherapy for 27 patients, double-combination therapy for 29 patients, and triple-combination therapy for 16 patients. Eleven deaths were registered among 28 patients who initially received PAH monotherapy. A significant decrease in survival was registered in patients treated with PAH-specific monotherapy compared with double-combination therapy (Figure 10A). Initial monotherapy was associated with a significant increase in mortality in Group III patients with low VRR [risk ratio (RR) 5.89; HR 1.06–32.6, *p* = 0.04].

**Figure 10:**
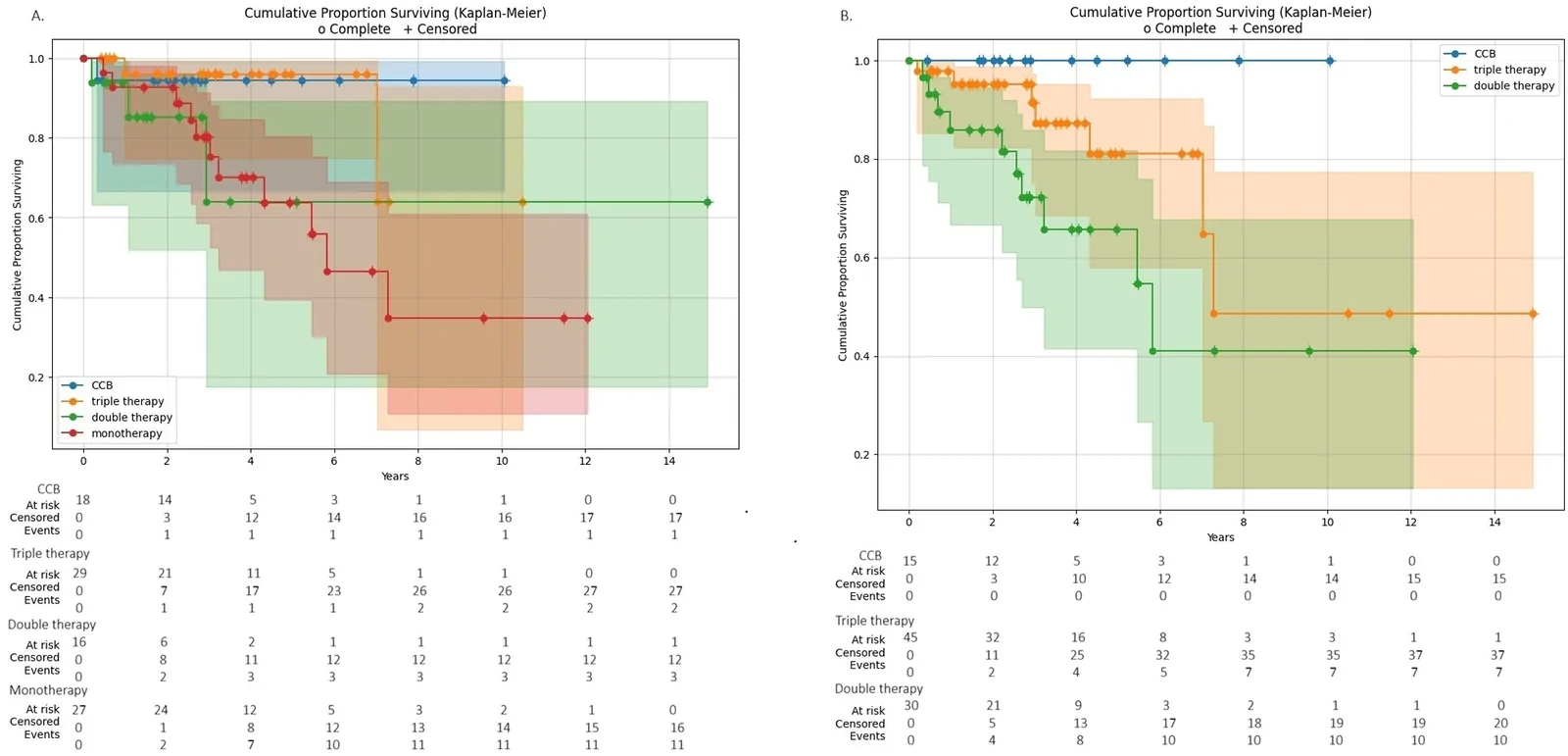
Survival in entire cohort of IPAH patients (*n* = 90) depending on initial therapy **(A)** and therapy at the time of analysis **(B)**.

### Therapy at the time of analysis

All patients initially treated with initial monotherapy underwent escalation of PAH-specific therapy. Thirty patients were on double-combination PAH specific therapy and 45 patients received triple-combination therapy at the time of analysis. Ten and seven patients died in the double-combination and triple-combination therapy groups, respectively. There was no difference in survival between the CCB and triple-combination therapy groups. Double-combination therapy was associated with higher mortality in comparison with triple-combination therapy (Figure 10B).

### CCB

One death occurred among 18 patients initially treated with CCBs. Two surviving patients experienced worsening of PAH with loss of positive AVT. PAH-specific therapy was prescribed and CCBs were withdrawn at follow-up.

Group III patients with ΔmPAP < 15% undergoing triple-combination therapy at follow-up demonstrated significantly better survival outcomes compared with double-combination therapy (*p* = 0.027) (Figure 11).

**Figure 11:**
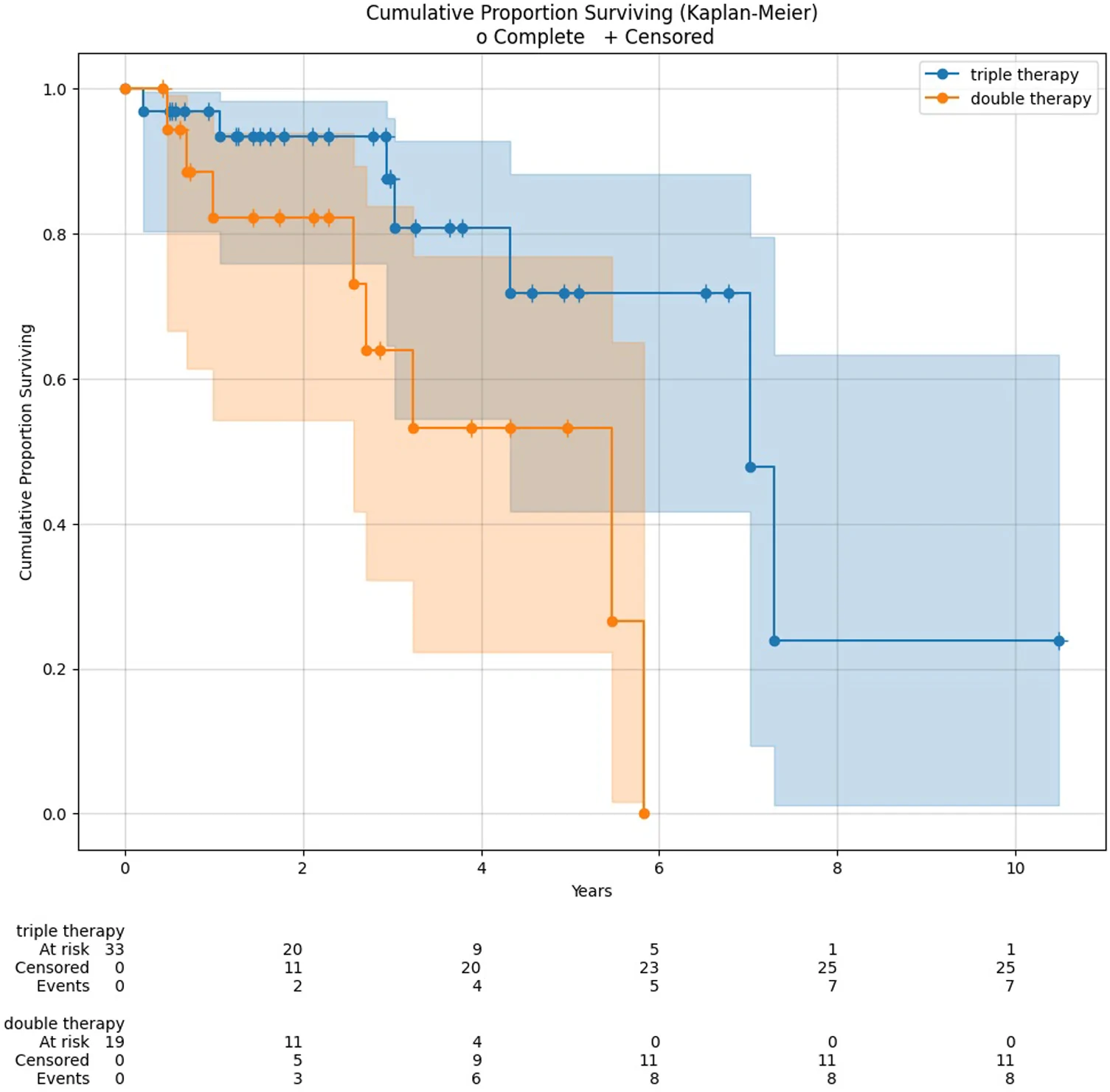
Survival in group III patients depending on double- versus triple-combination PAH therapy at the time of analysis.

We performed a separate analysis in 63 AVT-negative patients. No significant differences were registered between alive and dead patients in ΔmPAP in AVT in alive and dead patients [3.0 (1.0; 7.0) vs. 3.0 (1.5; 6) mmHg, *p* = 0.2], %ΔmPAP [5.8% (2.4%; 12.9%) vs. 4.58% (2.4%; 10.8%), *p* = 0.5) and %ΔPVR [17.9% (9.0%; 29.5%) vs. 14.2% (6.7%; 27.2%), *p* = 0.7), respectively. Correlation between %ΔmPAP and 2022 ESC/ERS risk status was not evaluated due to the lack of low-risk patients (*n* = 5). A trend toward better survival (log-rank test, *p* = 0.06) was observed in AVT-negative patients receiving triple-combination PAH-specific therapy during the follow-up compared with double-PAH therapy.

### Predictors of death

Significant correlations were identified between survival and PAH FC (WHO) [RR 2.38 (HR 1.14–4.9), *p* = 0.02], NT-proBNP ≥ 1,100 pg/mL [RR 4.1 (HR 1.47–11.5), *p* = 0.007], the degree of Δ%mPAP [RR 5.02 (HR 1.58–15.9), *p* = 0.006], degree of Δ%PVR [RR 2.15 (HR 1.14–4.1), *p* = 0.02], cardiac index [RR 0.32 (HR 0.12–0.81), *p* = 0.02], PAC during AVT [RR 0.36 (HR 0.17–0.76), *p* = 0.007], ΔPAC [RR 0.1 (HR 0.013–0.88), *p* = 0.037], RVEDD/LVEDD ratio on Echo [RR 9.8 (HR 2.4–39.2), *p* = 0.001], pericardial effusion [RR 3.3 (HR 1.2–9.0), *p* = 0.02], systolic TAVs [RR 0.7 (HR 0.55–0.88), *p* = 0.003], ESC 2022 risk score [RR 4.8 (HR 1.6–14.4), *p* = 0.005], ESC risk category [RR 3.45 (HR 1.5–7.8), *p* = 0.003], and follow-up therapy [RR 0.41 (HR 0.21–0.78), *p* = 0.007] in univariate analysis (Table 2; Supplementary Table 2).

**Table 2:** Predictors of survival in IPAH patients (*n* = 90) according to univariate analysis (supplements).

Parameter	Beta	St. Error	Beta 95% lower	Beta 95% upper	t-value	Wald-Stat.	*p*	Risk ratio	Risk ratio 95% lower	Risk ratio 95% upper
Demography and comorbidity										
Prevalence	−0.783088	1.032041	−2.80585	1.239675	−0.758776	0.575741	0.447992	0.456993	0.060455	3.454492
Age ≥ 60 years	0.984361	0.594700	−0.181229	2.149951	1.655223	2.739764	0.097889	2.676101	0.834244	8.584441
Gender	0.084052	0.644671	−1.17948	1.347585	0.130380	0.016999	0.896266	1.087686	0.307439	3.848119
BMI ≥ 30, kg/m^2^	−0.251272	0.578459	−1.38503	0.882488	−0.434381	0.188687	0.664015	0.777811	0.250316	2.416905
Arterial hypertension	−0.052950	0.494776	−1.02269	0.916793	−0.107019	0.011453	0.914775	0.948427	0.359625	2.501256
Ischemic heart disease	0.431742	1.045642	−1.61768	2.481162	0.412896	0.170483	0.679686	1.539937	0.198359	11.95515
COPD	0.104990	1.045486	−1.94413	2.154104	0.100422	0.010085	0.920010	1.110699	0.143112	8.620168
Diabetes	0.198481	0.581815	−0.941854	1.338817	0.341142	0.116378	0.732999	1.219549	0.389904	3.814528
Chronic kidney disease	1.324298	0.769487	−0.183868	2.832464	1.721014	2.961890	0.085258	3.759544	0.832045	16.98727
Thyroid disease	0.875340	0.535530	−0.174280	1.924960	1.634529	2.671684	0.102158	2.399690	0.840061	6.854873
Smoking	0.721600	0.647875	−0.548211	1.991412	1.113796	1.240541	0.265375	2.057724	0.577983	7.325871
Functional class and exercise tolerance										
Edema	0.720241	0.488521	−0.237241	1.677724	1.474332	2.173656	0.140402	2.054929	0.788801	5.353358
Syncope	−0.196987	0.494104	−1.16541	0.771439	−0.398676	0.158942	0.690135	0.821201	0.311794	2.162876
FC (WHO)	**0** **.** **866715**	**0** **.** **376529**	**0** **.** **128731**	**1** **.** **604699**	**2** **.** **301853**	**5** **.** **298526**	**0** **.** **021350**	**2** **.** **379082**	**1** **.** **137384**	**4** **.** **976361**
6MWD, m	−0.003518	0.001921	−0.007283	0.000248	−1.83066	3.351328	0.067160	0.996489	0.992743	1.000248
VO_2_ peak, mL/kg/min	−**0.198665**	**0** **.** **079111**	−**0.353720**	−**0.043610**	−**2.51122**	**6** **.** **306222**	**0** **.** **012036**	**0** **.** **819824**	**0** **.** **702071**	**0** **.** **957327**
VO_2_peak%Pred	−0.053865	0.017156	−0.087491	−0.020239	−3.13965	9.857386	**0** **.** **001693**	0.947560	0.916227	0.979964
VO_2_ peak < 15 mL/kg/min	−2.35198	1.098751	−4.50549	−0.198467	−2.14059	4.582139	**0** **.** **032314**	0.095181	0.011048	0.819987
VO_2_ AT, mL/kg/min	−**0.292709**	**0** **.** **105397**	−**0.499284**	−**0.086134**	−**2.77719**	**7** **.** **712800**	**0** **.** **005486**	**0** **.** **746239**	**0** **.** **606965**	**0** **.** **917472**
O_2_/HR	0.002665	0.051883	−0.099025	0.104355	0.051359	0.002638	0.959040	1.002668	0.905720	1.109994
O_2_/HR % Predicted	−0.034620	0.012789	−0.059685	−0.009555	−2.70709	7.328342	**0** **.** **006791**	0.965972	0.942061	0.990491
VE/VCO2, slope	0.126240	0.034231	0.059149	0.193332	3.687894	13.60056	**0** **.** **000226**	1.134555	1.060933	1.213285
PetCO_2_ rest, KPa	−**1.82720**	**0** **.** **669978**	−**3.14033**	−**0.514067**	−**2.72725**	**7** **.** **437916**	**0** **.** **006390**	**0** **.** **160863**	**0** **.** **043268**	**0** **.** **598058**
PetCO_2_ peak, KPa	−**1.67026**	**0** **.** **538076**	−**2.72487**	−**0.615647**	−**3.10413**	**9** **.** **635611**	**0** **.** **001910**	**0** **.** **188199**	**0** **.** **065555**	**0** **.** **540291**
Laboratory										
NT-proBNP, pg/mL	0.000179	0.000067	0.000048	0.000309	2.679496	7.179697	**0** **.** **007377**	1.000179	1.000048	1.000309
NT-proBNP ≥ 1,100 pg/mL	**1** **.** **411342**	**0** **.** **524506**	**0** **.** **383329**	**2** **.** **439355**	**2** **.** **690803**	**7** **.** **240422**	**0** **.** **007132**	**4** **.** **101457**	**1** **.** **467161**	**11** **.** **46564**
Creatinine, µmol/L	0.026398	0.013240	0.000448	0.052348	1.993802	3.975245	**0** **.** **046182**	1.026749	1.000448	1.053742
eGFR, mL/min·1.73 m^2^	−0.018653	0.010937	−0.040089	0.002782	−1.70561	2.909114	0.088090	0.981519	0.960704	1.002786
Hemoglobin, g/L	0.009841	0.012434	−0.014530	0.034213	0.791464	0.626415	0.428679	1.009890	0.985575	1.034805
C-reactive protein, mg/L	0.165602	0.072461	0.023580	0.307624	2.285382	5.222970	**0** **.** **022297**	1.180104	1.023860	1.360190
SatO_2_, %	−0.090612	0.065537	−0.219062	0.037839	−1.38260	1.911575	0.166798	0.913373	0.803272	1.038564
Hemodynamics										
AVT positive status	**2** **.** **435774**	**1** **.** **040366**	**0** **.** **396694**	**4** **.** **474854**	**2** **.** **341266**	**5** **.** **481526**	**0** **.** **019225**	**11** **.** **42465**	**1** **.** **486901**	**87** **.** **78178**
mPAP, mmHg	0.026990	0.016703	−0.005746	0.059727	1.615935	2.611245	0.106118	1.027358	0.994270	1.061546
mPAP in AVT ≤ 40 mmHg	−0.915204	0.548829	−1.99089	0.160481	−1.66756	2.780747	0.095413	0.400435	0.136574	1.174076
ΔmPAP, %	−0.057052	0.022241	−0.100645	−0.013460	−2.56513	6.579893	**0** **.** **010318**	0.944545	0.904254	0.986630
ΔmPAP: >30%; 30–15%; <15%	**1** **.** **613002**	**0** **.** **589751**	**0** **.** **457110**	**2** **.** **768894**	**2** **.** **735053**	**7** **.** **480514**	**0** **.** **006240**	**5** **.** **017850**	**1** **.** **579503**	**15** **.** **94099**
PCWP, mmHg	0.005007	0.068199	−0.128660	0.138674	0.073416	0.005390	0.941476	1.005019	0.879273	1.148750
PAP, mmHg	0.037794	0.052193	−0.064503	0.140091	0.724121	0.524351	0.468997	1.038518	0.937534	1.150379
CI, L/min/m^2^	−**1.14863**	**0** **.** **481969**	−**2.09327**	−**0.203987**	−**2.38320**	**5** **.** **679640**	**0** **.** **017169**	**0** **.** **317071**	**0** **.** **123283**	**0** **.** **815473**
SV index, mL/min/m^2^	−0.084565	0.031011	−0.145345	−0.023785	−2.72697	7.436360	**0** **.** **006395**	0.918911	0.864723	0.976495
PVR, WU	0.072168	0.024772	0.023615	0.120721	2.913246	8.487002	**0** **.** **003579**	1.074836	1.023896	1.128310
ΔPVR,%	−0.038696	0.014656	−0.067422	−0.009970	−2.64023	6.970802	**0** **.** **008289**	0.962043	0.934801	0.990079
ΔPVR:1 > 30%; 2–15–30%; 3 < 15%	**0** **.** **766769**	**0** **.** **324034**	**0** **.** **131674**	**1** **.** **401865**	**2** **.** **366323**	**5** **.** **599484**	**0** **.** **017972**	**2** **.** **152800**	**1** **.** **140737**	**4** **.** **062768**
PAC, mL/mmHg	−1.07007	0.598625	−2.24336	0.103211	−1.78755	3.195334	0.073858	0.342984	0.106102	1.108725
PAC in AVT, mL/mmHg	−**1.02671**	**0** **.** **385107**	−**1.78150**	−**0.271908**	−**2.66602**	**7** **.** **107671**	**0** **.** **007679**	**0** **.** **358185**	**0** **.** **168385**	**0** **.** **761924**
ΔPAC, mL/mmHg	−**2.24923**	**1** **.** **081302**	−**4.36854**	−**0.129913**	−**2.08011**	**4** **.** **326852**	**0** **.** **037524**	**0** **.** **105481**	**0** **.** **012670**	**0** **.** **878172**
ΔPAC, %	−0.017361	0.008797	−0.034603	−0.000118	−1.97343	3.894434	**0** **.** **048455**	0.982789	0.965989	0.999881
Pulmonary function test										
FVC, %	0.001508	0.015280	−0.028440	0.031456	0.098692	0.009740	0.921383	1.001509	0.971961	1.031956
FEV_1_, %	0.006305	0.015226	−0.023538	0.036148	0.414072	0.171456	0.678824	1.006325	0.976737	1.036809
DLCO, %	−0.055183	0.018010	−0.090482	−0.019884	−3.06398	9.387962	**0** **.** **002186**	0.946312	0.913490	0.980313
Echocardiography (*n* = 90) and MRI (*n* = 53)										
RA square, mm/m^2^	0.045746	0.027313	−0.007785	0.099278	1.674919	2.805355	0.093960	1.046809	0.992245	1.104373
RVEDD/LVEDD ratio	**2** **.** **279359**	**0** **.** **709321**	**0** **.** **889116**	**3** **.** **669602**	**3** **.** **213438**	**10** **.** **32618**	**0** **.** **001313**	**9** **.** **770411**	**2** **.** **432977**	**39** **.** **23630**
Pericardial effusion	**1** **.** **183866**	**0** **.** **518453**	**0** **.** **167717**	**2** **.** **200015**	**2** **.** **283458**	**5** **.** **214179**	**0** **.** **022410**	**3** **.** **266979**	**1** **.** **182601**	**9** **.** **025152**
TAPSE, mm	−**0.129401**	**0** **.** **056679**	−**0.240490**	−**0.018311**	−**2.28302**	**5** **.** **212195**	**0** **.** **022436**	**0** **.** **878622**	**0** **.** **786242**	**0** **.** **981856**
TAVs, cm/s	−**0.358509**	**0** **.** **120186**	−**0.594069**	−**0.122949**	−**2.98295**	**8** **.** **897978**	**0** **.** **002857**	**0** **.** **698717**	**0** **.** **552076**	**0** **.** **884309**
TAPSE/SPAP, mm/mmHg	−5.19906	3.187575	−11.4466	1.048471	−1.63104	2.660290	0.102892	0.005522	0.000011	2.853286
RVESVi, mL/m^2^	0.021174	0.014375	−0.007001	0.049348	1.472954	2.169594	0.140773	1.021399	0.993024	1.050586
RVEF, %	−0.093392	0.056158	−0.203459	0.016676	−1.66302	2.765635	0.096318	0.910837	0.815903	1.016816
SV index (MRI), mL/m^2^	0.039738	0.043459	−0.045440	0.124915	0.914378	0.836087	0.360525	1.040538	0.955577	1.133052
ESC/ERS 2022 3-strata risk stratification and therapy										
ESC 2022 score	**1** **.** **574097**	**0** **.** **559156**	**0** **.** **478171**	**2** **.** **670023**	**2** **.** **815129**	**7** **.** **924953**	**0** **.** **004879**	**4** **.** **826380**	**1** **.** **613122**	**14** **.** **44030**
ESC risk category: 1-low; 2-intermediate; 3-high	**1** **.** **238453**	**0** **.** **415439**	**0** **.** **424208**	**2** **.** **052698**	**2** **.** **981073**	**8** **.** **886795**	**0** **.** **002875**	**3** **.** **450273**	**1** **.** **528379**	**7** **.** **788888**
Initial therapy: 1-mono; 2-double; 3- triple PAH therapy; 4-CCB	−0.434121	0.276146	−0.975358	0.107116	−1.57207	2.471399	0.115945	0.647834	0.377057	1.113063
Follow-up therapy: 1-doule; 2-triple PAH therapy; 3-CCB	−**0.897952**	**0** **.** **335490**	−**1.55550**	−**0.240404**	−**2.67654**	**7** **.** **163865**	**0** **.** **007442**	**0** **.** **407403**	**0** **.** **211084**	**0** **.** **786310**

6MWT, 6-minute walk test; AVT, acute vasoreactive testing; BMI, body mass index; CCB, calcium channel blockers; CI, cardiac index; COPD, Chronic obstructive pulmonary disease; DLCO, diffusion capacity of the lungs for carbon monoxide; RVEDD/LVEDD, the ratio of the end-diastolic dimension of the right ventricle to the end-diastolic dimension of the left ventricle; lateral E/e’ratio, the ratio of early diastolic mitral inflow velocity to early diastolic lateral mitral annulus velocity; eGFR, estimated glomerular filtration rate (the estimated GFR was calculated according to the CKD-EPI equation); FC, functional class; FEV_1_, forced expiratory volume in one second; FVC, forced vital capacity; HR, heart rate; LVEDD, left ventricular end-diastolic dimension; mPAP, mean pulmonary artery pressure; NT-proBNP, N-terminal fragment of B-type brain natriuretic peptide; PAC, pulmonary arterial compliance; PET CO_2_, peak, peak end-tidal carbon dioxide pressure; PVR, pulmonary vascular resistance; RA, right atrial; RAP, right atrial pressure; RFEF, right ventricular ejection fraction; RVESV, right ventricular end systolic volume; SatO_2_, arterial blood saturation; SVi, stroke volume index; SvO_2_, mixed venous blood saturation; TAPSE, tricuspid annulus plane systolic excursion; TAVs, systolic tricuspid annular velocity; VE/VCO_2_, the ratio of minute ventilation to carbon dioxide production;_;_VO_2_ peak, peak oxygen consumption; V′O_2_/HR, oxygen pulse. Bold values indicate statistically significant parameters (*p* < 0.05).

Several independent predictors of survival were identified: the degree of Δ%mPAP [HR 5.99 (1.37–26.2), *p* = 0.017], NT-proBNP > 1,100 pg/mL [HR 8.1 (1.67–39.2), *p* = 0.009], and the number of PAH-specific therapies at follow-up [HR 0.07 (0.018–0.3), *p* = 0.0003]. The ESC/ERS 2022 three-strata scale lost its predictive significance during multivariate analysis (Table 3).

**Table 3:** Independent predictors of survival in IPAH patients (*n* = 90) according to multivariate analysis.

Parameter	Parameter estimate	Standard error	Chi-square	*p*-value	95% lower CL	95% upper CL	Hazard ratio	95% hazard ratio lower CL	95% hazard ratio upper CL
FC (WHO)	0.70156	0.677188	1.07328	0.300206	−0.62570	2.02882	2.016898	0.534885	7.60514
NT-proBNP ≥ 1,100 pg/mL	**2** **.** **09292**	**0** **.** **804538**	**6** **.** **76724**	**0** **.** **009285**	**0** **.** **51605**	**3** **.** **66978**	**8** **.** **108547**	**1** **.** **675402**	**39** **.** **24343**
ΔmPAP: 1, >30%; 2–15, 30%; 3, <15%	**1** **.** **79093**	**0** **.** **753130**	**5** **.** **65481**	**0** **.** **017408**	**0** **.** **31482**	**3** **.** **26704**	**5** **.** **995044**	**1** **.** **370019**	**26** **.** **23362**
ESC/ERS risk category: 1, low; 2, intermediate; 3, high	−0.44656	0.616355	0.52493	0.468745	−1.65460	0.76147	0.639823	0.191169	2.14142
Follow-up therapy: 1, double; 2, triple PAH therapy; 3, CCB	−**2.57886**	**0** **.** **727895**	**12** **.** **55215**	**0** **.** **000396**	−**4.00551**	−**1.15221**	**0** **.** **075860**	**0** **.** **018215**	**0** **.** **31594**

CCB, calcium channel blockers; FC, functional class; PAH, pulmonary arterial hypertension. Bold values indicate statistically significant parameters (*p* < 0.05).

### Comparison of standard 2022 ESC/ERS three-strata scale and the experimental risk stratification scale

The investigated parameter for the VRR assessment, %∆mPAP improved survival prognostification of the ESC/ERS three-strata risk stratification scale. Results of the bootstrap superiority test were (0.27, 0.54). The upper bound exceeding 0.50 suggests that, in a subset of resamples, the inclusion of the experimental feature improved predictive performance. Without the experimental feature (Δ%mPAP), the model achieved the following performance: MAPE = 9.82%; log-rank test (high–intermediate risk): *p* = 0.02684028415382257; log-rank test (intermediate–low risk): *p* = 0.02209228945050308; log-rank test (high–low risk): *p* = 0.028189067833756683; C-index = 0.7510548523206751. With the inclusion of the experimental feature (Δ%mPAP), performance was as follows: MAPE = 9.66%; log-rank test (high–intermediate risk): *p* = 0.02684028415382257; log-rank test (intermediate–low risk): *p* = 0.02209228945050308; log-rank test (high–low risk): *p* = 0.028189067833756683; C-index = 0.7552742616033755 (Figure 12).

**Figure 12:**
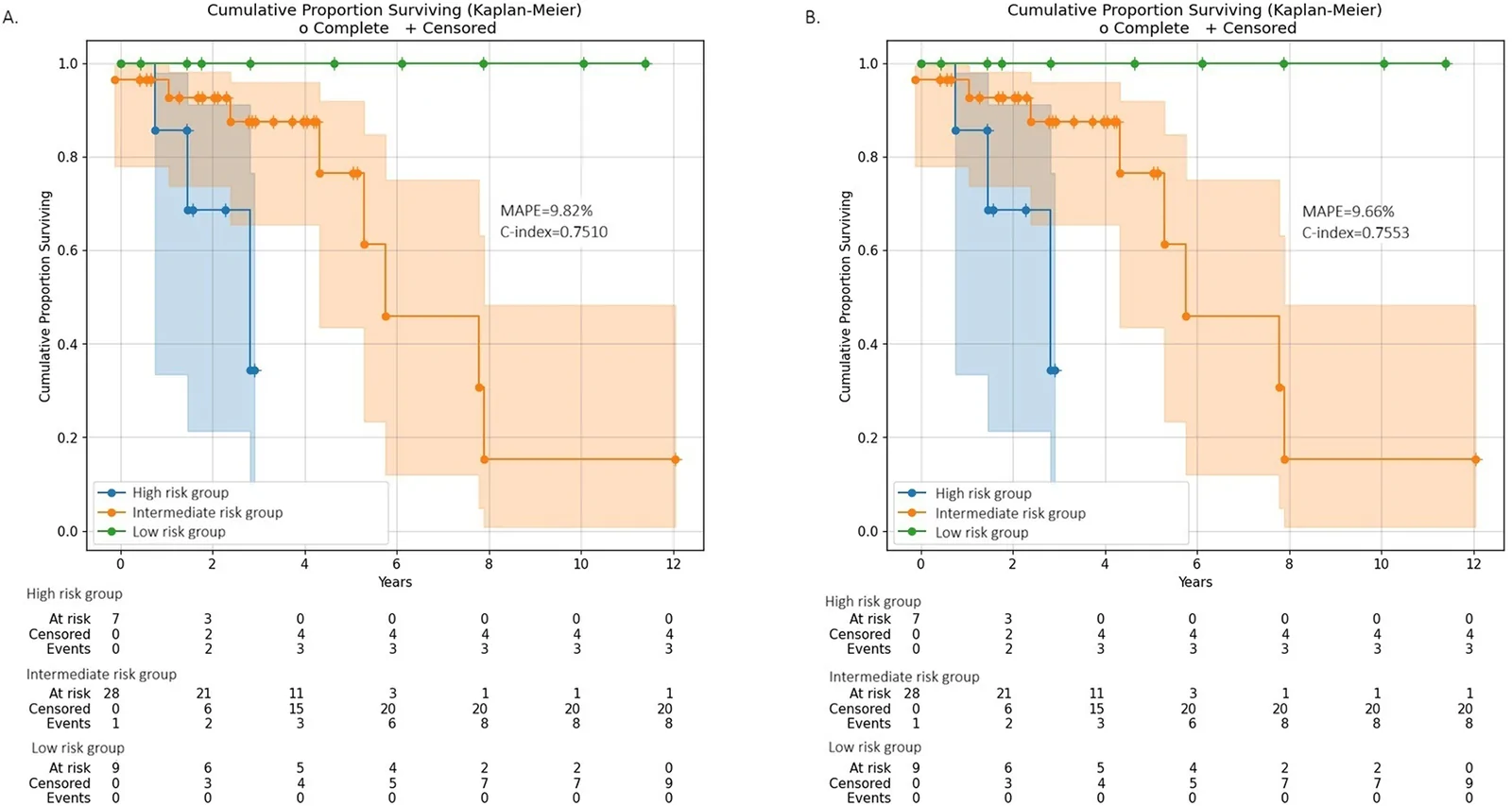
Modeling of survival prediction using the standard 2022 ESC/ERS three-strata risk scale **(A)** and experimental modified ESC/ERS three-strata risk scale with pulmonary vasoreactivity feature **(B)**.

We assessed the hierarchical prognostic value of parameters included in both the 2022 ESC/ERS three-strata score and the experimental risk stratification scale in our cohort of IPAH patients. We confirmed well-known correlations between the established ESC/ERS risk scale parameters and confirmed a positive correlation between Δ%mPAP during AVT and survival (Figure 13).

**Figure 13:**
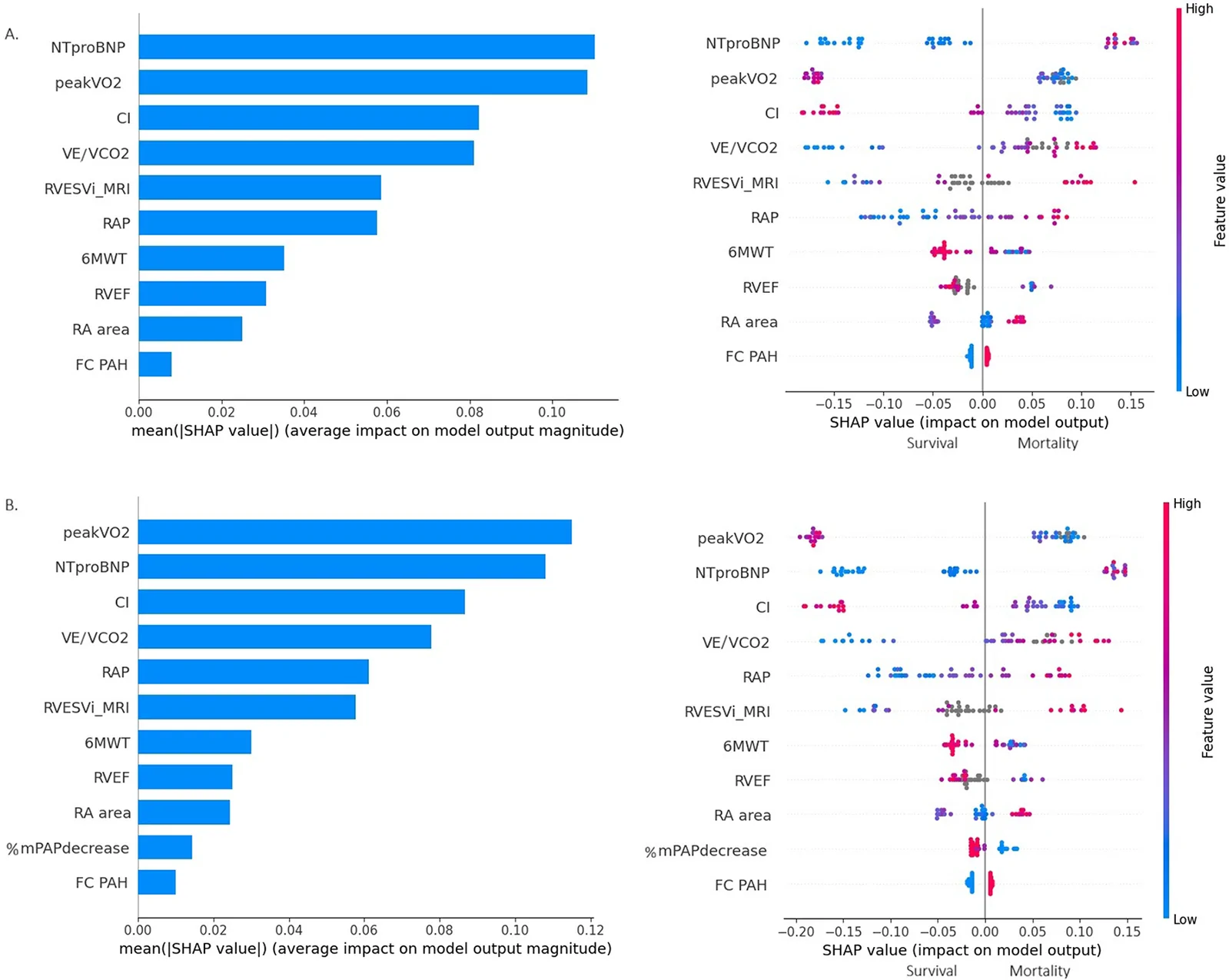
Hierarchical value and correlations with survival in evaluated models for the 2022 ESC/ERS three-strata risk scale parameters **(A)** and experimental risk stratification scale parameters **(B)**.

## Discussion

Various definitions have been applied to describe VRR (15–17). Absolute values of mPAP and PVR decrease during AVT, percentage changes from baseline, and combinations with each other and cardiac output have been used to characterize pulmonary VRR. The study of Maihotra et al. (15) demonstrated a reduction in mortality by 55% (OR 0.45, CI 0.22–0.96; *p* = 0.038) with the ΔmPAP ≥ 12% and by 53% (OR 0.47, CI 0.23–0.99; *p* = 0.047) for ΔPVR ≥ 30% in AVT in 80 patients group I PAH (15). Leuchte et al. (16) investigated VRR using inhaled iloprost in 66 IPAH patients with negative AVT (16). The authors classified VRR into four categories: ΔPVR in AVT ≤−10%, >−10 ≤ −20%, >−20 < −30%, and ≥−30%. A decrease in PVR <30% during AVT was associated with a 4.6-fold increase in the risk of mortality (CI 1–20.6, *p* < 0.05) in univariate analysis. Sun et al. (17) evaluated hemodynamics in AVT with inhaled iloprost in 487 incident patients with IPAH and negative AVT (38.18 ± 17.57 years, 171 men). The mean age of the study population was comparable to our cohort; however, there was a high proportion of males (35%) with lower SatO_2_ and higher PCWP. No data were presented regarding smoking status, lung function tests, and comorbidities (17). The authors identified a threshold of ΔPVR > 0.25 WU as associated with better survival. However, the prognostic significance of such a small reduction in PVR remains questionable.

We selected the mPAP decrease during AVT as a measure of the pulmonary vasoreactivity. Since the determination of mPAP is associated with the least error. Determining PVR requires a number of hemodynamic parameters, including cardiac output, which is itself a calculated value. Mean PAP averaging and the inaccuracy in PCWP estimation multiply errors in PVR calculation. Moreover, PVR is directly dependent on blood viscosity, meaning PVR values might differ in anemic or polycythemic patients (24). The other important reason for prioritizing ΔmPAP was the true correlation between a decrease in mPAP during PAH treatment and right-heart remodeling and survival outcomes. Indeed, in IPAH patients with positive AVT, the mPAP decrease below 37 mmHg was associated with successful CCB therapy (1). Ishiguro et al. (25) registered the 10-year survival rate of 80% of PAH patients who achieved mPAP <42 mmHg with PAH-specific therapy, whereas the survival rate was only 25% in patients maintaining mPAP >42 mmHg (25).

The proposed %ΔmPAP for defining pulmonary vasoreactive reserve was an exploratory indicator rather than a validated one. The choice of Δ%mPAP cut-off values was based on mathematician modeling and the significant difference in Δ%mPAP observed across low-, intermediate-, and high-risk 2022 ESC/ERS three-strata categories [32.4 (12.3; 42.2) vs. 12.9 (3.01; 28.2) vs. 4.8% (1.8; 8.9), *p* = 0.0009]. Patients with high VRR had better FC PAH (WHO), physical performance, hemodynamic profiles, and right-heart remodeling characteristics. We did not register significant differences in demography and comorbidity between the three groups of Δ%mPAP. Strict inclusion criteria limited the number of elderly patients with comorbidities in our study. This may have influenced the relatively high percentage (30%) of patients with positive AVT according to conventional Sitbon criteria compared with other studies (1, 4, 12, 26, 27). The use of iloprost could be another reason for the large number of patients with positive AVT. The greatest hemodynamic effect was observed in AVT with inhaled iloprost in comparative studies with nitric oxide, sildenafil (28, 29), and intravenous adenosine (30). The low percentage (6%–8%) of true responders to CCB, loss of positive AVT in half of the patients, and a further decrease in survival served as the rationale for studying VRR in a mixed population of patients with positive and negative AVT.

We observed a strong positive correlation between ΔmPAP and both PAD and PAC. We noted a significant positive correlation between PAC and PAD. These data confirmed the close relationship between the pulmonary artery's capacity for vasodilation and stiffness as determined by MRI. Jardim et al. (31) showed that PAD of 10% differentiated IPAH patients with positive versus negative AVT with 100% sensitivity and 56% specificity. However, the study population was quite small (*n* = 19), and survival was not assessed (31). We did not find a significant association between PAD and survival, as only 4 deaths were registered among 53 patients who underwent MRI. Variable PAD values have been associated with survival in PAH patients in other studies (23, 32). The prognostic cut-off value for PAD needs further investigation in IPAH patients.

PAC describes the change in blood volume per unit change in pressure. PAC reflects the elastic properties of the pulmonary vasculature, which enable it to accommodate the stroke volume and attenuate the propagation of pulse pressure into the distal pulmonary arteries (33). In our study, patients with ΔmPAP <15% had the lowest ΔPAC during AVT. Patients with ΔmPAP >30% had the highest ΔPAC during AVT (1.5 mL/mmHg), comparable with the values obtained in long-term vasoresponders in a study by Gerhardt et al. (4). Cheng et al. (34) and Gerhardt et al. (4) identified PAC during AVT as a predictor of long-term response to CCB therapy and favorable prognosis (4, 34). In our study, PAC during AVT and ΔPAC were associated with survival in univariate regression analysis. DesJardin et al. (35) did not confirm additional prognostic value of PAC in survival prediction modeling (35).

CPET parameters have been well defined for non-invasive risk stratification and survival prediction (36, 37). We found significant correlations between Δ%mPAP and both VO_2_ peak and VE/VCO_2_. Our observation confirmed the physiological mechanism of oxygen consumption through pulmonary microvasculature recruitment and PVR decrease during physical exertion (38, 39). Further prospective studies are needed to evaluate PAD and CPET parameters in non-invasive VRR prediction (40, 41).

Risk stratification using the 2022 ESC/ERS three-strata scale significantly discriminated low- from the high-risk patients, but failed to dichotomize intermediate- versus low-risk and intermediate- versus high-risk patients. We noted significant differences in survival between the group with high VRR and both groups with intermediate and low VRR. Almost all deceased patients exhibited low VRR with ΔmPAP <15% during AVT. In our study, the degree of Δ%mPAP [HR 5.99 (1.37–26.2), *p* = 0.017], NT-proBNP > 1,100 pg/mL [HR 8.1 (1.67–39.2), *p* = 0.009], and the number of PAH-specific therapies at follow-up [HR 0.07 (0.018–0.3), *p* = 0.0003] were the only independent predictors of death. We applied mathematical modeling to compare the standard 2022 ESC/ERS three-strata risk stratification scale and the experimental ESC/ERS three-strata-based model with Δ%mPAP inclusion. The experimental 2022 ESC/ERS three-strata risk stratification scale improved stratification value when compared with the standard 2022 ESC/ERS three-strata approach (MAPE = 9.66%; C-index = 0.7552742616033755 vs. MAPE = 9.82%; C-index = 0.7510548523206751). The hierarchical value of Δ%mPAP was not high in comparison with other well-defined prognostic parameters, such as CPET, NT-proBNP, hemodynamics, and right-heart size and function.

No significant difference between alive and dead (*n* = 16, 25.3%) patients with negative AVT was registered in terms of ∆mPAP, ∆PVR and ∆PAC. The same results were observed by Leuchte et al. (16) in 66 IPAH patients with negative AVT. The absolute values of ΔmPAP did not differ between living and deceased (24.2%) patients (6.14 ± 0.85 vs. 4.2 ± 1.4 mmHg) (16). The monomorphic patient population with a low number of low-risk (7.9%) patients, the limited presence of elderly patients with comorbidities, and the overall small sample size may explain the lack of consistency in ΔmPAP and ΔPVR between living and deceased patients with negative AVT in our study. A trend toward better survival (log-rank test, *p* = 0.06) was observed in AVT-negative patients receiving triple-combination PAH-specific therapy during the follow-up compared with double-PAH therapy.

Significantly better survival was observed in patients with the triple-combination PAH therapy at follow-up when compared with double-combination therapy in the entire cohort, particularly in patients with the lowest VRR. Importantly, the vast majority of IPAH patients had low VRR with a decrease in mPAP <15%, corresponding to 2.5 mmHg (1; 4.5). Only 9.6% patients were classified as low risk in a group with low VRR. The majority of studies and meta-analyses demonstrated positive effects on hemodynamics (42) and survival with triple-combination PAH therapy in intermediate- to high-risk IPAH patients (13, 43–45). We could not establish direct causal relationship between Δ%mPAP and the PAH-specific therapy number due to the observational nature of the study, the small patient population, differences in treatment approaches, and drug availability during the investigated period. We did not register any significant difference in hemodynamics between incident and prevalent patients. The small number of prevalent patients (*n* = 10) in our study may have influenced the result. However, Leuchte et al. (16) reported no significant difference in hemodynamics in AVT with inhaled Iloprost between naive and pretreated IPAH patients (16). According to Naranjo et al. (46), positive AVT was observed in patients receiving PAH therapy with the same frequency as noted in previously published registry studies (46). No data exist regarding the influence of PAH-specific therapy on VRR. Further studies on pulmonary vasoreactivity and the treatment effects of pulmonary vasodilators are needed, given the emergence of sotatercept, which has a fundamentally different, anti-remodeling effect on pulmonary vasculature (47).

## Conclusion

The vast majority of patients exhibited low pulmonary vasoreactivity during AVT.

A significant association was confirmed between the percentage of mPAP decrease and pulmonary artery compliance, pulmonary artery distensibility, and CPET parameters.

The degree of Δ%mPAP, NT-proBNP >1,100 pg/mL, and the number of PAH-specific therapies at follow-up were the independent predictors of survival.

Prospective observational studies with a large number of patients are needed to confirm clinical utility of VRR in risk stratification and in guiding PAH therapy.

### Limitations

The present study has several limitations. The definition of VRR has not been validated as yet. Strict inclusion criteria for the study excluded patients with comorbidities, due to which we could not evaluate their potential influence on Δ%mPAP during AVT. The small number of patients and the observational nature of the study limited our ability to investigate the effect of PAH therapy on VRR.

The small number of patients also constrained the experimental modeling used to compare the prognostic value of the standard 2022 ESC/ERS three-strata risk scale with the experimental scale incorporating Δ%mPAP.

## Data Availability

The original contributions presented in the study are included in the article/Supplementary Material; further inquiries can be directed to the corresponding author.

## Footnotes

^1^RAMP is a continuously incrementing protocol (increase of work rate, e.g., every 2–15 s) to symptom-limited maximum of exercise is used as standard. This offers the advantage of a short protocol with low initial work rate and a brief duration of highintensity cardiopulmonary exercise. (Glaab, T., Taube, C. Practical guide to cardiopulmonary exercise testing in adults. Respir Res. 23, 9 (2022). https://doi.org/10.1186/s12931-021-01895-6)
