# Supplementary material for: Pulmonary vasoreactive reserve in patients with idiopathic pulmonary arterial hypertension: characteristics and clinical significance
Source: Front Cardiovasc Med. 2026 Oct 14;13:1893751. doi: 10.3389/fcvm.2026.1893751 (PMC13714976; doi:10.3389/fcvm.2026.1893751)
Supplement: Supplementary file 1 [file Table1.docx]

**Table 2** Predictors of survival in IPAH patients (n=90) according to univariative analysis (Supplements)

| **Parameter** | **Beta** | **St.Error** | **Beta 95%lower** | **Beta 95% upper** | **t-value** | **Wald-Stat.** | **p** | **Risk ratio** | **Risk ratio 95% lower** | **Risk ratio 95% upper** |
| --- | --- | --- | --- | --- | --- | --- | --- | --- | --- | --- |
| **Demography and comorbidity** | | | | | | | | | | |
| Prevalence | -0,783088 | 1,032041 | -2,80585 | 1,239675 | -0,758776 | 0,575741 | 0,447992 | 0,456993 | 0,060455 | 3,454492 |
| Age≥60 years | 0,984361 | 0,594700 | -0,181229 | 2,149951 | 1,655223 | 2,739764 | 0,097889 | 2,676101 | 0,834244 | 8,584441 |
| Gender | 0,084052 | 0,644671 | -1,17948 | 1,347585 | 0,130380 | 0,016999 | 0,896266 | 1,087686 | 0,307439 | 3,848119 |
| BMI≥30, kg/m^2^ | -0,251272 | 0,578459 | -1,38503 | 0,882488 | -0,434381 | 0,188687 | 0,664015 | 0,777811 | 0,250316 | 2,416905 |
| Arterial hypertension | -0,052950 | 0,494776 | -1,02269 | 0,916793 | -0,107019 | 0,011453 | 0,914775 | 0,948427 | 0,359625 | 2,501256 |
| Ischemic heart disease | 0,431742 | 1,045642 | -1,61768 | 2,481162 | 0,412896 | 0,170483 | 0,679686 | 1,539937 | 0,198359 | 11,95515 |
| COPD | 0,104990 | 1,045486 | -1,94413 | 2,154104 | 0,100422 | 0,010085 | 0,920010 | 1,110699 | 0,143112 | 8,620168 |
| Diabetes | 0,198481 | 0,581815 | -0,941854 | 1,338817 | 0,341142 | 0,116378 | 0,732999 | 1,219549 | 0,389904 | 3,814528 |
| Chronic kidney disease | 1,324298 | 0,769487 | -0,183868 | 2,832464 | 1,721014 | 2,961890 | 0,085258 | 3,759544 | 0,832045 | 16,98727 |
| Thyroid disease | 0,875340 | 0,535530 | -0,174280 | 1,924960 | 1,634529 | 2,671684 | 0,102158 | 2,399690 | 0,840061 | 6,854873 |
| Smoking | 0,721600 | 0,647875 | -0,548211 | 1,991412 | 1,113796 | 1,240541 | 0,265375 | 2,057724 | 0,577983 | 7,325871 |
| **Functional class and exercise tolerance** | | | | | | | | | | |
| Edema | 0,720241 | 0,488521 | -0,237241 | 1,677724 | 1,474332 | 2,173656 | 0,140402 | 2,054929 | 0,788801 | 5,353358 |
| Syncope | -0,196987 | 0,494104 | -1,16541 | 0,771439 | -0,398676 | 0,158942 | 0,690135 | 0,821201 | 0,311794 | 2,162876 |
| **FC (WHO)** | **0,866715** | **0,376529** | **0,128731** | **1,604699** | **2,301853** | **5,298526** | **0,021350** | **2,379082** | **1,137384** | **4,976361** |
| 6MWD, m | -0,003518 | 0,001921 | -0,007283 | 0,000248 | -1,83066 | 3,351328 | 0,067160 | 0,996489 | 0,992743 | 1,000248 |
| **VO_2_ peak, ml/kg/min** | **-0,198665** | **0,079111** | **-0,353720** | **-0,043610** | **-2,51122** | **6,306222** | **0,012036** | **0,819824** | **0,702071** | **0,957327** |
| VO_2_peak%Pred | -0,053865 | 0,017156 | -0,087491 | -0,020239 | -3,13965 | 9,857386 | **0,001693** | 0,947560 | 0,916227 | 0,979964 |
| VO_2_ peak<15 ml/kg/min | -2,35198 | 1,098751 | -4,50549 | -0,198467 | -2,14059 | 4,582139 | **0,032314** | 0,095181 | 0,011048 | 0,819987 |
| **VO_2_ AT, ml/kg/min** | **-0,292709** | **0,105397** | **-0,499284** | **-0,086134** | **-2,77719** | **7,712800** | **0,005486** | **0,746239** | **0,606965** | **0,917472** |
| O_2_/HR | 0,002665 | 0,051883 | -0,099025 | 0,104355 | 0,051359 | 0,002638 | 0,959040 | 1,002668 | 0,905720 | 1,109994 |
| O_2_/HR % Predicted | -0,034620 | 0,012789 | -0,059685 | -0,009555 | -2,70709 | 7,328342 | **0,006791** | 0,965972 | 0,942061 | 0,990491 |
| VE/VCO2, slope | 0,126240 | 0,034231 | 0,059149 | 0,193332 | 3,687894 | 13,60056 | **0,000226** | 1,134555 | 1,060933 | 1,213285 |
| **PetCO_2_ rest,** KPa | **-1,82720** | **0,669978** | **-3,14033** | **-0,514067** | **-2,72725** | **7,437916** | **0,006390** | **0,160863** | **0,043268** | **0,598058** |
| **PetCO_2_ peak,** KPa | **-1,67026** | **0,538076** | **-2,72487** | **-0,615647** | **-3,10413** | **9,635611** | **0,001910** | **0,188199** | **0,065555** | **0,540291** |
| **Laboratory** | | | | | | | | | | |
| NTproBNP, pg/ml | 0,000179 | 0,000067 | 0,000048 | 0,000309 | 2,679496 | 7,179697 | **0,007377** | 1,000179 | 1,000048 | 1,000309 |
| **NTproBNP≥1100 pg/ml** | **1,411342** | **0,524506** | **0,383329** | **2,439355** | **2,690803** | **7,240422** | **0,007132** | **4,101457** | **1,467161** | **11,46564** |
| Creatinin, µmol/l | 0,026398 | 0,013240 | 0,000448 | 0,052348 | 1,993802 | 3,975245 | **0,046182** | 1,026749 | 1,000448 | 1,053742 |
| eGFR, ml/min*1.73m^2^ | -0,018653 | 0,010937 | -0,040089 | 0,002782 | -1,70561 | 2,909114 | 0,088090 | 0,981519 | 0,960704 | 1,002786 |
| Hemoglobin, g/l | 0,009841 | 0,012434 | -0,014530 | 0,034213 | 0,791464 | 0,626415 | 0,428679 | 1,009890 | 0,985575 | 1,034805 |
| C-reactive protein, mg/l | 0,165602 | 0,072461 | 0,023580 | 0,307624 | 2,285382 | 5,222970 | **0,022297** | 1,180104 | 1,023860 | 1,360190 |
| SatO_2_, % | -0,090612 | 0,065537 | -0,219062 | 0,037839 | -1,38260 | 1,911575 | 0,166798 | 0,913373 | 0,803272 | 1,038564 |
| **Hemodynamics** | | | | | | | | | | |
| **AVT positive status** | **2,435774** | **1,040366** | **0,396694** | **4,474854** | **2,341266** | **5,481526** | **0,019225** | **11,42465** | **1,486901** | **87,78178** |
| mPAP, mmHg | 0,026990 | 0,016703 | -0,005746 | 0,059727 | 1,615935 | 2,611245 | 0,106118 | 1,027358 | 0,994270 | 1,061546 |
| mPAP in AVT ≤40 mmHg | -0,915204 | 0,548829 | -1,99089 | 0,160481 | -1,66756 | 2,780747 | 0,095413 | 0,400435 | 0,136574 | 1,174076 |
| ∆ mPAP, % | -0,057052 | 0,022241 | -0,100645 | -0,013460 | -2,56513 | 6,579893 | **0,010318** | 0,944545 | 0,904254 | 0,986630 |
| **∆ mPAP: >30%; 30-15%; <15%** | **1,613002** | **0,589751** | **0,457110** | **2,768894** | **2,735053** | **7,480514** | **0,006240** | **5,017850** | **1,579503** | **15,94099** |
| PCWP, mmHg | 0,005007 | 0,068199 | -0,128660 | 0,138674 | 0,073416 | 0,005390 | 0,941476 | 1,005019 | 0,879273 | 1,148750 |
| PAP, mmHg | 0,037794 | 0,052193 | -0,064503 | 0,140091 | 0,724121 | 0,524351 | 0,468997 | 1,038518 | 0,937534 | 1,150379 |
| **CI, l/min/m^2^** | **-1,14863** | **0,481969** | **-2,09327** | **-0,203987** | **-2,38320** | **5,679640** | **0,017169** | **0,317071** | **0,123283** | **0,815473** |
| SV index, ml/min/m^2^ | -0,084565 | 0,031011 | -0,145345 | -0,023785 | -2,72697 | 7,436360 | **0,006395** | 0,918911 | 0,864723 | 0,976495 |
| PVR, WU | 0,072168 | 0,024772 | 0,023615 | 0,120721 | 2,913246 | 8,487002 | **0,003579** | 1,074836 | 1,023896 | 1,128310 |
| ∆ PVR,% | -0,038696 | 0,014656 | -0,067422 | -0,009970 | -2,64023 | 6,970802 | **0,008289** | 0,962043 | 0,934801 | 0,990079 |
| **∆ PVR:1>30%; 2-15-30%; 3<15%** | **0,766769** | **0,324034** | **0,131674** | **1,401865** | **2,366323** | **5,599484** | **0,017972** | **2,152800** | **1,140737** | **4,062768** |
| PAC, ml/mmHg | -1,07007 | 0,598625 | -2,24336 | 0,103211 | -1,78755 | 3,195334 | 0,073858 | 0,342984 | 0,106102 | 1,108725 |
| **PAC in AVT, ml/mmHg** | **-1,02671** | **0,385107** | **-1,78150** | **-0,271908** | **-2,66602** | **7,107671** | **0,007679** | **0,358185** | **0,168385** | **0,761924** |
| **∆ PAC_,_ ml/mmHg** | **-2,24923** | **1,081302** | **-4,36854** | **-0,129913** | **-2,08011** | **4,326852** | **0,037524** | **0,105481** | **0,012670** | **0,878172** |
| ∆ PAC, % | -0,017361 | 0,008797 | -0,034603 | -0,000118 | -1,97343 | 3,894434 | **0,048455** | 0,982789 | 0,965989 | 0,999881 |
| **Pulmonary function test** | | | | | | | | | | |
| FVC, % | 0,001508 | 0,015280 | -0,028440 | 0,031456 | 0,098692 | 0,009740 | 0,921383 | 1,001509 | 0,971961 | 1,031956 |
| FEV_1,_ % | 0,006305 | 0,015226 | -0,023538 | 0,036148 | 0,414072 | 0,171456 | 0,678824 | 1,006325 | 0,976737 | 1,036809 |
| DLCO, % | -0,055183 | 0,018010 | -0,090482 | -0,019884 | -3,06398 | 9,387962 | **0,002186** | 0,946312 | 0,913490 | 0,980313 |
| **Echocardiography (n=90) and MRI (n=53)** | | | | | | | | | | |
| RA square, mm/m^2^ | 0,045746 | 0,027313 | -0,007785 | 0,099278 | 1,674919 | 2,805355 | 0,093960 | 1,046809 | 0,992245 | 1,104373 |
| **RVEDD/LVEDD ratio** | **2,279359** | **0,709321** | **0,889116** | **3,669602** | **3,213438** | **10,32618** | **0,001313** | **9,770411** | **2,432977** | **39,23630** |
| **Pericardial effusion** | **1,183866** | **0,518453** | **0,167717** | **2,200015** | **2,283458** | **5,214179** | **0,022410** | **3,266979** | **1,182601** | **9,025152** |
| **TAPSE, mm** | **-0,129401** | **0,056679** | **-0,240490** | **-0,018311** | **-2,28302** | **5,212195** | **0,022436** | **0,878622** | **0,786242** | **0,981856** |
| **TAVs, cm/s** | **-0,358509** | **0,120186** | **-0,594069** | **-0,122949** | **-2,98295** | **8,897978** | **0,002857** | **0,698717** | **0,552076** | **0,884309** |
| TAPSE/SPAP, mm/mmHg | -5,19906 | 3,187575 | -11,4466 | 1,048471 | -1,63104 | 2,660290 | 0,102892 | 0,005522 | 0,000011 | 2,853286 |
| RVESVi, ml/m^2^ | 0,021174 | 0,014375 | -0,007001 | 0,049348 | 1,472954 | 2,169594 | 0,140773 | 1,021399 | 0,993024 | 1,050586 |
| RVEF, % | -0,093392 | 0,056158 | -0,203459 | 0,016676 | -1,66302 | 2,765635 | 0,096318 | 0,910837 | 0,815903 | 1,016816 |
| SV index (MRI), ml/m^2^ | 0,039738 | 0,043459 | -0,045440 | 0,124915 | 0,914378 | 0,836087 | 0,360525 | 1,040538 | 0,955577 | 1,133052 |
| **ESC/ERS 2022 3-strata risk stratification and therapy** | | | | | | | | | | |
| **ESC 2022 score** | **1,574097** | **0,559156** | **0,478171** | **2,670023** | **2,815129** | **7,924953** | **0,004879** | **4,826380** | **1,613122** | **14,44030** |
| **ESC risk category: 1-low; 2-intermediate; 3-high** | **1,238453** | **0,415439** | **0,424208** | **2,052698** | **2,981073** | **8,886795** | **0,002875** | **3,450273** | **1,528379** | **7,788888** |
| Initial therapy: 1-mono; 2-double; 3- triple PAH therapy; 4-CCB | -0,434121 | 0,276146 | -0,975358 | 0,107116 | -1,57207 | 2,471399 | 0,115945 | 0,647834 | 0,377057 | 1,113063 |
| **Follow-up therapy: 1-doule; 2-triple PAH therapy; 3-CCB** | **-0,897952** | **0,335490** | **-1,55550** | **-0,240404** | **-2,67654** | **7,163865** | **0,007442** | **0,407403** | **0,211084** | **0,786310** |

**Footnote:** 6MWT, 6-minute walk test; AVT, acute vasoreactive testing; BMI, body mass index; CCB, calcium channel blockers; CI, cardiac index; DLCO, diffusion capacity of the lungs for carbon monoxide; RVEDD/LVEDD, the ratio of the end-diastolic dimension of the right ventricle to the end-diastolic dimension of the left ventricle; lateral E/e’ratio - the ratio of early diastolic mitral inflow velocity to early diastolic lateral mitral annulus velocity; eGFR, estimated glomerular filtration rate (the estimated GFR was calculated according to the CKD-EPI equation); FC, functional class; FEV_1_, forced expiratory volume in one second; FVC, forced vital capacity; HR, heart rate; LVEDD, left ventricular end-diastolic dimension; mPAP, mean pulmonary artery pressure; NT-proBNP, N-terminal fragment of B-type brain natriuretic peptide; PAC, pulmonary arterial compliance; PET CO_2_, peak, peak end-tidal carbon dioxide pressure; PVR, pulmonary vascular resistance; RA, right atrial; RAP, right atrial pressure; RF EF, right ventricular ejection fraction; RVESV, right ventricular end systolic volume; SatO_2_, arterial blood saturation; SVi, stroke volume index; SvO_2_, mixed venous blood saturation; TAPSE, tricuspid annulus plane systolic excursion; TAVs, *s*ystolic tricuspid annular velocity; VE/VCO_2 ,_,the ratio of minute ventilation to carbon dioxide production;_;_VO_2_ peak, peak oxygen consumption; V'O_2_/HR, oxygen pulse.
